# The Decade-Long Chinese Methadone Maintenance Therapy Yields Large Population and Economic Benefits for Drug Users in Reducing Harm, HIV and HCV Disease Burden

**DOI:** 10.3389/fpubh.2019.00327

**Published:** 2019-11-12

**Authors:** Lei Zhang, Xia Zou, Yong Xu, Nick Medland, Liwei Deng, Yin Liu, Shu Su, Li Ling

**Affiliations:** ^1^Sun Yat-sen Center for Migrant Health Policy, Department of Medical Statistics, School of Public Health, Sun Yat-sen University, Guangzhou, China; ^2^China-Australia Joint Research Center for Infectious Diseases, School of Public Health, Xi'an Jiaotong University Health Science Centre, Xi'an, China; ^3^Melbourne Sexual Health Centre, Alfred Health, Melbourne, VIC, Australia; ^4^Faculty of Medicine, Nursing and Health Sciences, Central Clinical School, Monash University, Melbourne, VIC, Australia; ^5^Department of Epidemiology and Biostatistics, College of Public Health, Zhengzhou University, Zhengzhou, China

**Keywords:** methadone maintenance treatment, human immunodeficiency virus, hepatitis C, mathematical model, economic benefit

## Abstract

**Objectives:** We aimed to conduct a comprehensive evaluation of the population impact of methadone maintenance treatment (MMT) for its future program planning.

**Methods:** We conducted a literature review of the effects of MMT in China on HIV and HCV disease burden, injecting, and sexual behaviors and drug-related harm during 2004–2015. Data synthesis and analysis were conducted to obtain the pooled estimates of parameters for a mathematical model which was constructed to evaluate the effectiveness and cost-effectiveness of the program.

**Results:** Based on a review of 134 articles, this study demonstrated that MMT is highly effective in reducing crime-related, high risk sexual, and injecting behaviors. The model estimated US$1,037 m which was invested in MMT from 2004 to 2015 has prevented 29,463 (15,325–43,600) new HIV infections, 130,563 (91,580–169,546) new HCV infections, 10,783 (10,380–11,187) deaths related to HIV, HCV and drug-related harm, and 338,920.0 (334,596.2–343,243.7) disability-adjusted life years (DALYs). The costs for each prevented HIV infection, HCV infection, death, and DALY were $35,206.8 (33,594.8–36,981.4), $7,944.7 ($7,714.4–8,189.2), $96,193.4 (92,726.0–99,930.2), and $3,060.6 ($3,022.0–3,100.1) respectively.

**Conclusion:** The Chinese MMT program has been effective and cost-effective in reducing injecting, injecting-related risk behaviors and adversities due to HIV/HCV infection and drug-related harm among drug users.

## Introduction

Sharing of injection equipment is the major risk factor for transmission of HIV and hepatitis C (HCV) among drug users (DUs) worldwide (1–3). HIV infection requires life-long treatment and treatment of HCV is expensive and of limited availability in all but the most resource-rich settings. Methadone maintenance treatment (MMT) is an evidence-based opioid substitution treatment for opiate addiction and injecting drug use. Evidence shows that MMT substantially reduces the frequency of drug use, sharing of injecting equipment and drug-related crimes (4, 5). In settings where drug use is closely related to commercial sex, MMT also reduces commercial sex work and high-risk sexual exposure and provides opportunities for health education, safe sex education, and condom programs in MMT CENTERS (6, 7). MMT is an effective intervention to reduce HIV and HCV transmission (8–10).

Despite numerous evidence supporting its effectiveness, MMT is not accepted equally around the world. For example, Russia banned MMT in 2013 (11), leaving an estimated 1.8 million injecting drug users (IDUs) in the country without treatment. Russian authorities are thought to have feared that MMT may promote more widespread drug use, although there is no evidence that it does. In other settings, although MMT is legal, uptake of it varies greatly. MMT is mostly voluntary in resource-rich settings, although compulsory treatment or so-called “mixed models” exist in many countries (12).

In China, estimated over three million “registered drug users” were known to the authority. The establishment of MMT in China formed a part of the national response to the epidemic of HIV in IDUs in the first decade of the new millennium. In 2004, the Chinese Ministry of Health and Ministry of Public Security piloted MMT in eight counties in China. By 2014, there were 765 MMT sites in 28 Chinese provinces and municipalities with 410,000 individuals in treatment (13, 14), making China's MMT program one of the largest harm reduction and comprehensive care programs in the world.

Despite the rapid and large-scale expansion of the program, concerns have been raised about its effectiveness, safety, and cost. Drop-out rates as high as 45% in the first year have been reported (15). Speculations about its efficacy and potential impacts on the overall DU population have been raised. From a health policy perspective, the sustainability of the program will depend on a measurable return on the large financial public investment. Quantifying the benefits in harm reduction programs is complex because it is impossible to know precisely what harm would have occurred if the program had not been present. Mathematical modeling is commonly used to measure the effect of MMT on HIV and HCV transmission (16–25).

We comprehensively reviewed available epidemiological and program data and modeled the effect of MMT programme on HIV and HCV transmission and drug-related harm. Based on which, this study aims to provide a timely assessment of the effectiveness and cost-effectiveness of the decade-long Chinese MMT program by assessing its role in harm reduction and prevention of HIV/HCV transmission with a mathematical model.

## Materials and Methods

### Literature Search

We conducted a comprehensive search of the peer-reviewed literature, government reports, and the gray literature for sources of parameter values (Table S6). We searched in the following five areas: (1) Epidemiological data (including IDU population sizes, MMT uptake and coverage and HIV and HCV prevalence, notifications, and incidence); (2) Behavioral data (including injecting and non-injecting drug users, sharing of injecting equipment, commercial sex work, sexual behavior, and condom use); (3) Biological data (including HIV and HCV transmission probabilities, death rates, clinical stage transition probabilities, and treatment effectiveness); (4) Disease burden data (including disability-adjusted of life-years (DALYs) due to drug use, and HIV/HCV infection and the impact of MMT and HIV/HCV treatment); (5) Medical cost data (including the cost of HIV and HCV treatment and the MMT program, in China). We defined “IDUs” as those have been injecting drugs in the past 6 months. Otherwise, they were regarded as “non-IDUs.”

We searched the following six electronic databases for documents dated up to 1st November 2015: Chinese National Knowledge Infrastructure, Chong Qing VIP Information, Wanfang database, Chinese Biomedical Literature Database, PubMed/Medline, and Google Scholar. Chinese government websites were searched for relevant reports. A study was included if it: (1) reported the parameters and indicators as previously described, (2) published in Chinese or English. We excluded: (1) conference proceedings, qualitative studies, news articles, and case reports; (2) studies with the smaller sample size if multiple studies from the same data source were available; (3) local reports where national reports of the same data exist. For HIV and HCV epidemic data, we only included national systematic reviews and meta-analyses studies that are representative of the epidemics nationally and excluded individual studies. Two independent investigators (XZ and YX) reviewed all records to determine eligibility.

We extracted relevant parameter values and conducted a data synthesis and analysis using random effect model to estimate its pooled proportions and 95% confidence intervals (CIs) (Tables S1–S5). For each parameter, if there was only one data source, we adapted the mean value and 95% CIs of the parameters, where they are available, directly from the literature. In the case of multiple data sources, we estimated the mean and CIs by weighting the mean and CIs of each source according to their corresponding sample size.

### Model Design

We modeled HIV and HCV transmission in DUs in China. The model accounted for four subgroups of DUs: IDUs or non-IDUs either in or not in MMT. DUs who have had injected drugs in the past 6 months were regarded as IDUs. Otherwise, they were non-IDUs. The model allowed individuals to enroll in or cease MMT and move between “injecting” and “not injecting” (Figure S1). We modeled four progression stages for HIV and HCV infection: (1) susceptible; (2) infected but undiagnosed; (3) diagnosed and untreated, and (4) on treatment (or post-treatment for curative HCV treatment). Combining potential co-infections resulted in 16 possible HIV/HCV disease stages, which were applied to each drug use subgroup. In this model, HIV and HCV were transmitted through contaminated injecting equipment, and HIV can also be sexually transmitted. There was no natural clearance for HIV infection, but 15–45% of HCV infected individuals cleared the infection spontaneously (26, 27). People living with HIV have access to antiretroviral therapy, while only interferon-based treatments for HCV were available in resource-limited settings, including China. The sustained virological response (SVR) rate among HCV infected patients who were treated with interferon-gamma is about 60% (28). Chronically infected individuals can progress to liver fibrosis, cirrhosis and hepatocellular carcinoma (HCC) (29, 30). The details of the disease progression were illustrated (Figure S2). We hypothesed that behavior of MMT participants at enrolment (baseline) would be similar to that of DUs in the community.

### Model Calibration

We calibrated the model to available epidemiological data. We used a Latin Hypercube sampling method to sample collected indicators within their uncertainty bounds and repeated 10,000 times. For each simulation, we estimated the goodness of fit between the output results and observed data based on the standard deviation of errors. Simulated outputs were ranked according to their goodness of fit, and the highest ranked 5% were chosen to generate the model outputs with 95% confidence intervals [CIs (31–33)].

### Effectiveness and Cost-Effectiveness Evaluation

The model generated epidemiological indicators for HIV and HCV in both DUs and IDUs and predicted these indicators in the absence of MMT in counterfactual scenarios in 2005–2015. These include prevalence, incidence, the estimated number of new infections and diagnoses, the number of individuals on treatment, drug-related deaths, and HIV/HCV related deaths. The differences in these indicators by comparing MMT (status quo) and “no MMT” (hypothetical) scenarios represented the effectiveness of MMT in China. We hence calculated the number of HIV and HCV cases prevented, drug use person-years prevented, HIV and HCV related deaths prevented, and drug-related deaths prevented because of MMT. The number of HIV-, HCV- and harm-related disability adjusted life years (DALYs, Table S3) prevented were calculated based on simulated results and health utilities in each disease stage among DUs in China. We estimated the total MMT investment over the past decade and calculated the amount for each DALY prevented by reducing HIV, HCV, and harm in the Chinese DUs, respectively. The unit cost for drug use and HIV/HCV treatment was listed in Table S4. We used 3% discounting rate for cost and DALY in our economic analysis. The scenarios are considered as “cost-effective” if the cost required to prevent one DALY was below three times China's per capita GDP ($8,100 in 2016).

### Uncertainty Analysis

Uncertainty analysis was used to identify the variations in indicators and inform the accuracy of output results, by describing the distribution, central, and discrete tendency of the outputs. We used descriptive statistics to describe the distribution and uncertainties of model outputs.

### Software for Data Analysis

We used Matlab R2016a for the data analysis.

## Results

### MMT Program in China

**Figure 1:**
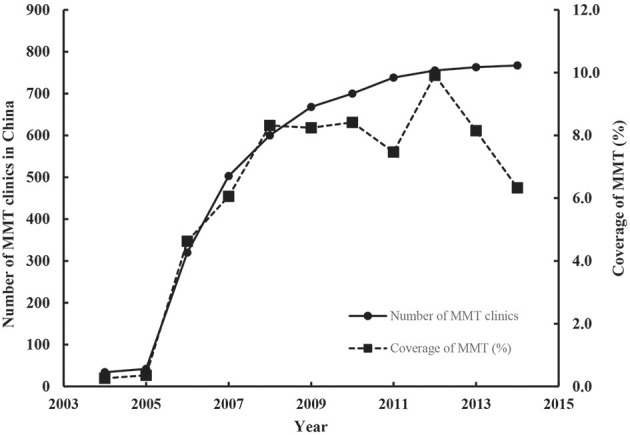
Temporal trend of number of MMT clinics and the coverage of MMT (%) in China, 2004–2014 (The number of MMT clinics was sourced from Annual Report on Drug Control in China, 2005–2015, whereas the coverage of MMT was calculated by dividing the number of individuals on MMT by the total number of registered DUs over these years).

According to the literature, between 2004 and 2015, China invested a total of US$1,037 million in the MMT program, and the number of MMT sites increased from 34 to 767. The number of individuals receiving MMT increased from ~3,000 in 2004, peaked at 208,000 in 2012, and then decreased to 187,000 in 2015, covering 6% of total registered Chinese DUs (Figure 1).

### MMT Effectively Reduced Risk Behaviors of DUs

**Figure 2:**
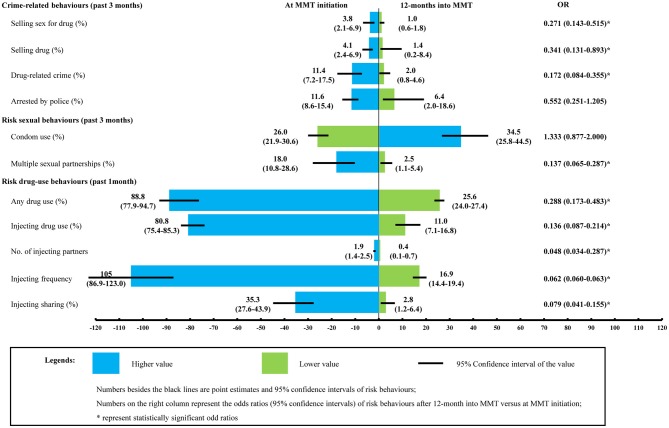
Comparison of risk behaviors in DUs at MMT enrolment and 12 months after treatment initiation. MMT entrants are regarded as a proxy of community DUs. Its comparison with MMT clients after 12-month treatment provides empirical evidence about how MMT may have changed DUs' risk behaviors. The two sets of parameters were subsequently fed into the model as input parameters to inform the calculation of HIV/HCV transmission probability [Data sources: a published meta-analysis (15) and MMT clinical database of Guangdong province, 2006–2014 (8)].

Our literature review identified 134 studies that provide parameter values for our model (see Tables S1–S5). Based on the literatures, we estimated that the proportion of individuals selling sex for drugs, selling drugs, and other drug-related crime in first 12 months after MMT initiation was reduced from 3.8% (2.1–6.9%) to 1.0 (0.6–1.8%), 4.1% (2.4–6.9%) to 1.4% (0.2–8.4%), and 11.4% (7.2–17.5%) to 2.0% (0.8–4.6%). respectively. Similarly, the proportion of individuals who reported multiple sexual partners, had used drugs and had injected was reduced from 18.0% (10.8–28.6%) to 2.5% (1.1–5.4%), 88.8% (77.9–94.7%) to 25.6% (24.0–27.4%), and from 80.8% (75.4–85.3%) to 11.0% (7.1–16.8%). Those who continued to inject did so infrequently, ~17 (14–19) times every 30 days with an average of 0.4 (0.1–0.7) injecting partners. The proportion of injecting sharing decreased from 35.3% (27.6–43.9%) to 2.8% (1.2–6.4%). The odds in conducting drug-related crimes, risk drug-use behaviors, and having multiple sexual partnerships were significantly lower in MMT clients 12 months into treatment than at enrolment (Figure 2).

### Comparatively Large Reduction in Drug-Related Harm Than in HIV/HCV

**Figure 3:**
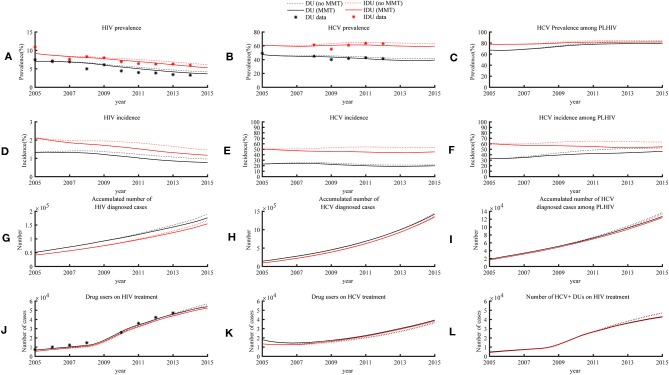
HIV and HCV epidemic trends among DUs and IDUs in the presence of MMT program (status quo) and absence of the program (hypothetical), 2005–2015.

The model showed that the impact of MMT on HIV prevalence was relatively small. The estimated HIV prevalence in 2015 among Chinese DUs in the absence of MMT was 4.2% (2.7–5.6%) and 6.2% (3.8–8.6%) for all DUs (including non-injecting) and IDUs, respectively. In the presence of MMT, these were only 0.5 and 0.8% lower, respectively (Figure 3A). Similarly, the model estimated HCV prevalence among all DUs and injecting users to be 41.9% (38.4–45.3%) and 63.4% (59.9–66.9%) in 2015 in the absence of MMT, only 2.2 and 4.2% higher than the current level in the presence of MMT (Figure 3B). A similar reduction in HCV prevalence (HIV+ DUs: 0.1%; HIV+ IDUs 0.5%) was also observed in HIV+ DUs (Figure 3C).

MMT has limited impacts on reducing new cases of HIV and HCV in the overall DU population. The model estimated HIV and HCV incidence among DUs in 2015 were 0.8% (0.4–1.2%) and 19.9% (16.8–23.0%), which was 0.2 and 1.7% lower than that in the absence of the program. In 2015, HIV and HCV incidence among IDUs were 1.2% (0.6–1.9%) and 45.4% (38.9–51.8%), corresponding to 0.3 and 7.2% reduction (Figures 3D,E). MMT had the largest impact on HCV incidence among DUs living with HIV. The estimated HCV incidence among all DUs and IDUs living with HIV in 2015 were 46.2% (41.3–50.8%) and 54.6% (50.6–58.2%), which were 6.3 and 8.8% lower than the scenario without MMT (Figure 3F). In the absence of the MMT program, 228,036 (141,697–314,375) and 2,667,156 (2,341,096–2,993,216) new HIV and HCV infections among DUs would have occurred over the decade to 2015. However, the national Chinese MMT program have reduced 29,463 (15,325–43,600) and 130,563 (91,580–169,546) new HIV and HCV infections among DUs. For those 410,000 individuals who ever participated in MMT from 2005, the program has accumulatively reduced 914,218 (813,927–1,014,510) person-years of drug consumption (Table 1).

**Table 1:** Evaluation of population impacts and cost-effectiveness of the Chinese MMT program during 2004–2015.

	**2004**	**2005**	**2006**	**2007**	**2008**	**2009**	**2010**	**2011**	**2012**	**2013**	**2014**	**2015**	**Total**	**Economic evaluation**	
**MMT program**															
Number of clinics	34	42	320	503	600	668	700	738	755	763	767	–	767		
MMT participants (×1000)	3.0	4.1	37.0	58.0	93.7	110.0	130.0	134.0	208.0	201.7	187.0	231.9	410.0		
MMT coverage (%)	0.3	0.4	4.6	6.1	8.3	8.2	8.4	7.5	9.9	8.2	6.3	–	6.3		
MMT spending (USD $m)	3.5	4.5	36.7	57.7	79.4	90.9	101.7	105.9	139.1	136.9	130.7	150.2	1037.3		
Mean MMT spending perperson-year ($)	1173.8	1089.5	993.0	994.5	847.9	826.5	782.1	790.0	668.7	678.5	699.2	647.9	2529.3		
**Population impacts**														**Cost/case averted (×1000)**	
HIV infections averted (×1000)	–	0.1 (0.1, 0.1)	0.3 (0.3, 0.4)	0.8 (0.7, 0.8)	1.3 (1.2, 1.3)	1.9 (1.8, 2)	2.6 (2.5, 2.7)	3.5 (3.3, 3.6)	4.4 (4.2, 4.6)	5.2 (4.9, 5.4)	5.7 (5.4, 6)	6.3 (5.9, 6.6)	29.5 (28.0, 30.9)	$35.2 (33.6, 37.0)	
HCV infections averted (×1000)	–	0 (0, 0)	0.1 (0, 0.1)	0.1 (0, 0.2)	0.2 (0.1, 0.4)	0.4 (0.1, 0.6)	0.6 (0.2, 0.9)	0.8 (0.3, 1.3)	1.1 (0.5, 1.7)	1.3 (0.6, 2)	1.6 (0.8, 2.3)	1.9 (1.1, 17.6)	130.6 (126.7, 134.5)	$7.9 (7.7, 8.2)	
Drug-use person-years averted (×1000)	–	6.3 (6.3, 6.4)	19.1 (18.9, 19.2)	36.6 (36.3, 37)	53.2 (52.7, 53.7)	72.4 (71.7, 73.1)	94.3 (93.3, 95.2)	117.7 (116.5, 119)	142.1 (140.5, 143.6)	150 (148.2, 151.8)	140.8 (138.9, 142.6)	135.6 (133.7, 137.4)	914.2 (904.2, 924.2)	$1.1 (1.1-1.2)	
HIV-related deaths averted	–	12 (12,13)	55 (54, 56)	149 (146, 152)	290 (283, 298)	459 (446, 473)	656 (634, 678)	906 (872, 939)	1194 (1146, 1242)	1510 (1444, 1576)	1822 (1737, 1907)	2128 (2023, 2234)	8305.8 (7965.3, 8646.4)	$124.9 (120.0, 130.2)	
HCV-related deaths averted	–	10 (10,10)	96 (96, 96)	354 (354, 354)	849 (849, 850)	1566 (1565, 1567)	2448 (2446, 2449)	3470 (3468, 3472)	4612 (4610, 4615)	5852 (5848, 5856)	6997 (6992, 7002)	7826 (7821, 7832)	793.5 (774.4, 812.6)	$1307.2 (1276.6, 1339.4)	$96.2 (92.7, 99.9)
Harm-related deaths averted	–	9 (9,9)	25 (25,26)	49 (48,49)	69 (68, 70)	92 (91, 94)	117 (114, 119)	141 (138, 144)	165 (161, 169)	165 (160, 170)	139 (134, 145)	117 (110, 123)	1043.3 (1018.1, 1068.5)	$994.2 (970.8, 1018.8)	
HIV-related DALY averted (×1000)	–	0 (0, 0)	0.2 (0.2, 0.2)	0.5 (0.5, 0.5)	1 (1,1)	1.6 (1.5, 1.6)	2.1 (2.1, 2.2)	2.9 (2.8, 3)	3.7 (3.6, 3.8)	4.6 (4.4, 4.7)	5.5 (5.2, 5.7)	6.8 (6.5, 7.1)	26.0 (25.1, 26.9)	39.9 (38.5, 41.3)	
HCV-related DALY averted (×1000)	–	0.1 (0.1, 0.1)	0.4 (0.4, 0.4)	0.9 (0.9, 0.9)	1.6 (1.6, 1.6)	2.5 (2.4, 2.5)	3.5 (3.4, 3.5)	4.6 (4.5, 4.7)	5.9 (5.7, 6)	7.1 (6.9, 7.2)	8 (7.8, 8.2)	8.7 (8.4, 8.9)	30.9 (30.1, 31.7)	33.6 (32.7, 34.4)	$3.1 (3.0, 3.1)
Harm-related DALY averted (×1000)	–	2.8 (2.8, 2.8)	8.3 (8.2, 8.3)	15.6 (15.5, 15.8)	22.2 (22, 22.5)	29.5 (29.2, 29.8)	37.2 (36.8, 37.6)	45 (44.5, 45.6)	52.7 (52.1, 53.4)	54 (53.3, 54.8)	49.1 (48.3, 49.8)	45.6 (44.8, 46.4)	344.1 (339.8, 348.3)	3.0 (3.0, 3.0)	
**Treatment cost**														**Benefit-cost ratio**	
Spending on HIV care/ treatment saved ($m)	–	−0.2 (−0.2,−0.1)	−0.6 (−0.6,−0.6)	−1.6 (−1.6,−1.5)	−2.6 (−2.7,−2.6)	−2.6 (−2.7,−2.5)	−1.1 (−1.3,−0.9)	0.9 (0.6, 1.2)	4.3 (3.8, 4.7)	8.9 (8.3, 9.6)	14.5 (13.6, 15.4)	20.4 (19.2, 21.7)	31.7 (28.4, 35.0)	$0.03 (0.03, 0.03)	
Spending on HCV care /treatment saved ($m)	–	0.1 (0.1, 0.1)	0.8 (0.8, 0.9)	3.0 (2.9, 3.1)	7.3 (7, 7.5)	13.9 (13.5, 14.4)	22.3 (21.6, 23)	31.5 (30.5, 32.6)	41.9 (40.4, 43.3)	52.9 (51, 54.8)	62.2 (59.9, 64.5)	68.4 (65.8, 71)	276.6 (267, 286.2)	$0.3 (0.3, 0.3)	$6.7 (6.6, 6.8)
Reduction in drug cost ($m)	–	48.2 (47.6, 48.7)	152 (150.3, 153.7)	298 (294.5, 301.5)	433 (427.6, 438.3)	576.4 (568.9, 583.8)	724.2 (714.2, 734.2)	875.1 (862.2, 888)	1024.5 (1008.3, 1040.8)	1049.5 (1031.1, 1067.9)	958 (939, 976.9)	900.1 (880.5, 919.6)	6683.1 (6578.5, 6787.7)	$6.4 (6.3, 6.5)	

### MMT Enhances the Diagnosis of HIV, HCV, and Subsequent Treatment

We estimated that in the presence of MMT, the number of HIV diagnoses was reduced by 13,327 (7,007–19,647) cases due to its reduced incidence (Figure 3G) and consequently 8,306 (7,965–8,646) HIV-related deaths were prevented. A similar trend was also observed in HIV/HCV coinfections, for which the number of newly diagnosed was reduced by 9,521 (5,517–13,525) cases (Figure 3I). In contrast, although the number of HCV diagnoses were reduced by 19,841 (1,793–37,889) cases (Figure 3H), the MMT program enables additional 16,422 (12,693–20,150) individuals to receive interferon-γ treatment for HCV (Figures 3J–L). In turn, this prevented 793 (774–813) HCV-related deaths. Estimated 1,043 (1,018–1,068) harm-related deaths were prevented, the total number of prevented deaths amounted to 10,783 (10,380–11,187) (Table 1).

### MMT Is Cost-Effective

On average, it costed $35,206.8 (33,594.8–36,981.4), $7,944.7 ($7,714.4–8,189.2) to prevent one HIV and HCV infection, and $1,134.6 ($1,122.3–1,147.2) to prevent 1 year of drug-consumption. Overall, the Chinese MMT program reduced DALY lost due to HIV, HCV and drug-related harm by $39,863.9 ($38,501.9–41,325.8), $33,570.4 ($32,740.4–34,443.7), $3,014.8 ($2,977.9–3,052.6), respectively. This amounts to 338,920.0 (334,596.2–343,243.7) DALYs for HIV, HCV, and drug-related harm combined and a cost of $3,060.6 ($3,022.0–3,100.1) for each DALY prevented.

We estimated that over the past decade, the Chinese MMT program had saved $0.03 ($0.03–0.03), $0.3 ($0.3–0.3), and $6.4 ($6.3–6.5) on HIV, HCV infection and drug-related harm for each dollar invested in the Chinese MMT.

## Discussion

Our study showed that MMT in China expanded rapidly from 2004 to 2012 and then declined slightly between 2012 and 2015. MMT was highly effective in reducing drug-related crime, sharing of contaminated injecting equipment, and high risk sexual behaviors. Although there was a positive impact of MMT on the HIV and HCV epidemics, the largest benefit came from the reduction in drug-related harm. Our model indicates MMT was a very cost-effective intervention to improve the overall health of Chinese DUs.

The decline in MMT coverage could be due to several possible reasons. Firstly, individuals leaving treatment early due to an economic burden or other causes could result in the decline. One study in Guangzhou identified that MMT clients frequently left treatment because they perceived that they were fully recovered from their addiction (34). Despite programs specifically targeting adherence and retention, up to 45% of new clients discontinue MMT in the first year (15, 35, 36). Secondly, there is evidence in China of a switch between the use of opiates to synthetic amphetamine-like substances (ALS) with a >6 times increase in some areas, which may render MMT less relevant to the needs of DUs (37). This may also be reflected in the older age of MMT users than ALS users (38). Thirdly, relaxation of compulsory treatment requirements may mean that individuals who had previously been coerced into treatment and treated against their will choose not to continue treatment when those restrictions were removed. Conversely, the fear of detention and involuntary treatment may prevent individuals who might otherwise wish to participate in MMT from doing so.

We argue that MMT should focus on harm reduction, while the provision of HIV and HCV care and treatment should continue to be strengthened. If considered only in terms of HIV and HCV prevention, the MMT program may not be cost-effective. However, when including the benefits from harm reduction as a whole, it is highly cost-effective. With the HIV prevalence has demonstrated substantial decline in the past and the current increasing proportion synthetic drug use that requires less or no injection, the incidence of HIV in DUs due to injection sharing is likely to decline further. The role of MMT for HIV prevention, as it was originally proposed, could be seen to be less important. In contrast, the role of MMT as a harm reduction program is becoming more important, both in the light of the results of our study and because other key harm reduction interventions are in decline. For example, needle and syringe exchange programs (NSP), which were once well-funded by the Global Funds for AIDS, tuberculosis and malaria, has been largely defunded and sites have not promoted needle exchange since 2012 (39). MMT is necessary as a harm reduction program for Chinese DUs, in fact is the only significant intervention program for these people. Therefore, providing an environment for individuals to reduce opioid drug use during and after cessation of MMT should be a priority for the program.

Voluntary MMT with a fixed term should be encouraged. In Australia, MMT clients are recommended to remain on treatment for at least 12 months to achieve enduring lifestyle changes (40). Six months of follow-up care is then offered to clients who choose to withdraw from MMT. In contrast, MMT clients are expected to remain on treatment for life in China. There is no “exit strategy” for clinicians to negotiate with their clients. A client is considered to have “dropped-out” if they do not pick up their medication for a consecutive 30 days. After leaving MMT, over 60% of clients resume drug use within 3 months (41–44). Notably, cycling in-and-out MMT is common in about 80% of MMT clients (44). Without a planned “exit strategy,” it is impossible to provide the necessary physical and psychological care to a DU when he decides to leave the program and face the challenges of abstinence in daily life. A well-planned and timed treatment cessation can serve as a starting point for additional interventions to focus on relapse prevention, including behavioral and psychological strategies to avoid being tempted back to their old circle of drug-using friends and a lifestyle of addiction. Alleviation of psychological aspects of addiction and re-integration into society requires the involvement of psychologists and social workers.

The future of MMT hinges on its capability to provide comprehensive interventions for DUs. Sexually transmitted and blood borne viral infections are now routinely screened for at MMT enrolment and regularly during the course of the treatment. Currently, HIV-positive clients are referred to medical institutions outside of the MMT program to receive treatment. Sustained engagement in MMT substantially increases the initiation of ART, and cross-referrals between ART and MMT services can often increase concurrent engagement (45). However, a recent pilot study in six MMT clinics of Sichuan province demonstrating that integration of ART into MMT sites is practical and feasible (46).

HCV is 6–8 times more prevalent than HIV among DUs, suggesting an even more urgent need for HCV care and treatment (47). Currently, most HCV is diagnosed passively in a hospital setting. In contrast within MMT programs, HCV screening is routinely conducted with over 90% coverage (47). Despite this, HCV-positive clients are referred back to hospitals for further treatment (47). We argue that MMT clinics are an ideal, controlled and feasible environment for HCV treatment provision. Treatment for 12 months with pegylated interferon and ribavirin is the standard of care for chronic HCV in China. Despite a 45% drop-out rate, the 12-month treatment is still achievable for a majority of DUs living with HCV. Direct-acting antivirals for treatment of genotype 1b HCV has been officially approved in China in 2017 (48). It will reduce treatment time and side effects in most patients. The oral medication can be co-administered with methadone. MMT can be a platform for HCV treatment that closes a gap in the continuum of HCV care and leads to better treatment outcomes.

Our study has several limitations. Firstly, we assumed a homogeneous HIV/HCV epidemiological and behavioral pattern in DUs across China, whereas geographical variations are known to occur, particularly in Southwest and Northwest China. Secondly, our model did not account for viral load and duration of HIV infection which may affect infectiousness. Thirdly, our estimate of MMT investment was based on the average spending of MMT clinics in China, while the actual spending may vary based on the size and number of clients in the clinics. Fourthly, the cost-effectiveness analysis was conducted from the perspective of governmental expenditure and did not include the economic loss to the community or, which if incorporated would have dramatically increased the cost-effectiveness measures.

## Conclusion

The Chinese MMT program has been effective and cost-effective in reducing injecting, injecting-related risk behaviors and adversities due to HIV/HCV infection and drug-related harm among drug users.

## Data Availability Statement

All datasets generated for this study are included in the article/**Supplementary Material**.

## Author Contributions

LL, LZ, and XZ conceived this work and finalized the manuscript. LZ and XZ contributed to the development of the manuscript. YX and XZ conduct the data collection and modeling. LD, YL, and SS contributed in data collection. NM polished the language throughout the manuscript. All authors read and approved the manuscript.

### Conflict of Interest

The authors declare that the research was conducted in the absence of any commercial or financial relationships that could be construed as a potential conflict of interest.
