# Supplementary material for: The Decade-Long Chinese Methadone Maintenance Therapy Yields Large Population and Economic Benefits for Drug Users in Reducing Harm, HIV and HCV Disease Burden
Source: Front Public Health. 2019 Nov 12;7:327. doi: 10.3389/fpubh.2019.00327 (PMC6861367; doi:10.3389/fpubh.2019.00327)
Supplement: Supplementary file 1 [file Data_Sheet_1.docx]

Supplementary Material

***Literature search***

To populate our model with real-life data, we conducted a comprehensive search of the peer-reviewed literature, government reports and documents and the grey literature for sources of parameter values. We conducted a literature search in the following five areas: 1. Epidemiological data (including injecting drug user population sizes, MMT uptake and coverage and HIV and HCV prevalence, notifications and incidence); 2. Behavioural data (including injecting and non-injecting drug use, sharing of injecting equipment, commercial sex work, sexual behaviour and condom use); 3.Biological data (including HIV and HCV transmission probabilities, death rates, clinical stage transition probabilities and treatment effectiveness); 4. Disease burden data (including disability-adjusted of life-years lost (DALYs) due to drug use, and HIV/HCV infection and the impact of MMT and HIV/HCV treatment); 5. Medical cost data (including the cost of HIV and HCV treatment and the MMT program, in China).

We searched the following six electronic databases for documents dated up to 1^st^ November 2015: Chinese National Knowledge Infrastructure (CNKI), Chong Qing VIP Information (CQVIP), Wanfang data, Chinese Biomedical Literature Database, PubMed/Medline, and Google Scholar. Chinese government websites were searched for government reports.

**Search strategies**：We used the combination of the following keywords (in English and Chinese) to conduct the literature search:

1. **Epidemiological data**
   1. injecting drug user population sizes, MMT uptake and coverage: *(“drug use” OR “people who inject drugs” OR “heroin use” OR “heroin addict” OR “methadone maintenance” OR “drug abuse” OR “heroin abuse” OR “opioid dependence” OR “opiate addicted” OR “opiate abuse”) AND (“population size” OR “scale” OR “coverage”) AND (“China” OR “Chinese”)*
   2. HIV and HCV prevalence, notification and incidence: *(“drug use” OR “people who inject drugs” OR “heroin use” OR “heroin addict” OR “methadone maintenance” OR “drug abuse” OR “heroin abuse” OR “opioid dependence” OR “opiate addicted” OR “opiate abuse”) AND (“human immunodeficiency virus” OR “acquired immunodeficiency syndrome” OR “HIV” OR “AIDS” OR "venereal disease" OR "sexually transmitted diseases" OR "sexually transmitted infections" OR “STD” OR “STI” OR “HCV” OR "hepatitis C") AND (“China” OR “Chinese”)*
2. **Behavioural data**
   1. injecting and non-injecting drug use, sharing of injecting equipment: *(“drug use” OR “people who inject drugs” OR “heroin use” OR “heroin addict” OR “methadone maintenance” OR “drug abuse” OR “heroin abuse” OR “opioid dependence” OR “opiate addicted” OR “opiate abuse”) AND (“risk behaviour” OR “risk behavior” OR “needle sharing” OR “syringe sharing”) AND (“China” or “Chinese”)*
   2. commercial sex work, sexual behaviour and condom use: *("sexual behaviour" OR “sexual behavior” OR "commercial sex" OR "condom use" OR "unprotected sex") AND*  *(“drug use” OR “people who inject drugs” OR “heroin use” OR “heroin addict” OR “methadone maintenance” OR “drug abuse” OR “heroin abuse” OR “opioid dependence” OR “opiate addicted” OR “opiate abuse”) AND (“China” OR “Chinese”)*
3. **Biological data**
   1. HIV and HCV transmission probabilities: *(“human immunodeficiency virus” OR “acquired immunodeficiency syndrome” OR “HIV” OR “AIDS” OR "venereal disease" OR "Sexually transmitted disease" OR "Sexually transmitted infection" OR “HCV” OR "hepatitis C") AND (“transmit” OR “transmission” OR “risk”) AND (“infectivity”)*
   2. death rates: *(“drug use” OR “people who inject drugs” OR “heroin use” OR “heroin addict” OR “methadone maintenance” OR “drug abuse” OR “heroin abuse” OR “opioid dependence” OR “opiate addicted” OR “opiate abuse”) AND (“mortality” OR “death” OR “survival”)*
   3. clinical stage transition probabilities and treatment effectiveness: *(“human immunodeficiency virus” OR “acquired immunodeficiency syndrome” OR “HIV” OR “AIDS” OR "venereal disease" OR "sexually transmitted diseases" OR "sexually transmitted infections" OR “STD” OR “STI” OR “HCV” OR "hepatitis C") AND (“progress” OR “progression” OR “transition”)*
4. **Burden of diseases**
   1. disability-adjusted of life-years lost (DALYs) due to drug use, and HIV/HCV infection and the impact of MMT and HIV/HCV treatment: *(“disability-adjusted life years” OR “DALY” OR “utility”) AND (“human immunodeficiency virus” OR “acquired immunodeficiency syndrome” OR “HIV” OR “AIDS” OR "venereal disease" OR "sexually transmitted diseases" OR "sexually transmitted infections" OR “STD” OR “STI” OR “HCV” OR "hepatitis C")*
5. **Medical cost**
   1. cost of HIV and HCV treatment: *(“human immunodeficiency virus” OR “acquired immunodeficiency syndrome” OR “HIV” OR “AIDS” OR "venereal disease" OR "sexually transmitted diseases" OR "sexually transmitted infections" OR “STD” OR “STI” OR “HCV” OR "hepatitis C") AND (“economic evaluation” or “cost”) AND (“China” OR “Chinese”)*
   2. cost of MMT program: *(“methadone maintenance treatment”) AND (“economic evaluation” or “cost”) AND (“China” OR “Chinese”)*

***Selection criteria:***

A study was included if it: (1) reported the parameters and indicators as previously described, (2) published in Chinese or English. We excluded: (1) conference proceedings, qualitative studies, news articles, and case reports; (2) if multiple studies from the same data source were available, we excluded the studies with the smaller sample size; (3) local reports where national reports of the same data exist. For HIV and HCV epidemic data, we only included national systematic reviews and meta-analyses studies are representative of the epidemics nationally and excluded individual studies. Two independent investigators (XZ and YX) reviewed all records to determine eligibility. If no study was found for a relevant model parameter, then the parameter was estimated by expert opinions.

***Data extraction and analysis***

We extracted relevant parameter values and conducted a data synthetic and analysis to estimate its best estimates and corresponding 95% confidence intervals (Table S1-4). For each parameter, if there was only one data source, we adapted the mean value and 95% confidence intervals (CIs) of the parameters, where they are available, directly from the literature. In the case of multiple data sources, we estimated the mean and CIs by weighting the mean and CIs of each source according to their corresponding sample size.

Epidemiological indicators in Table S1 are based on clinical data and used for the calibration of the epidemiological trend of HIV and HCV simulated by the model (Figure 3). We synthesised the parameters for risk sexual and injecting behaviour, HIV testing and treatment parameters in Chinese drug users in Table S2. We made several assumptions on behavioural parameters. Firstly, risk behaviours in drug users outside MMT did not change during the study period 2004-2014. Secondly, there is evidence that MMT significantly reduced drug-related risk behaviours (injecting acts and proportion of sharing) but rarely changed the sexual behaviours (times of sexual acts and condom use) (Figure 2). We assumed the changes in behaviours retain as long as the clients remain in MMT. Once they leave MMT, their risk behaviour again resembles those not on MMT. We have also included more detail description of time-varying of risk behaviours of drug users in MMT in Table S2. Values in Table S3-S4 provides information on life quality and treatment costs in each drug use and HIV and HCV infection stages.

***Model construction***

Our model consisted of two essential components. The first component accounts for the population movement of drug users in and out of MMT. We defined four subgroups of drug users: injecting or non-injecting drug user either in or not in MMT (Figure S1). The part of the model simulates both drug use and MMT initiation and termination. The second component of model accounts for the transmission and disease progression of both HIV and HCV in each of these subgroups. We use a deterministic compartmental approach to simulate the model. We describe these components in details as follows.

*Modelling population movement in and out MMT*

The schematic population flow of drug users between different subgroups is depicted in Figure S1. Non-IDU initiates injecting drug use with a transition rate $\varphi$. We use $\varphi_{NMMT}$ to represent the transition rate from non-IDU to IDU who are not on MMT, whereas $\varphi_{MMT}$ represents the transition rate from non-IDU to IDU on MMT. Similarly, we use $\omega_{NMMT}$ and $\omega_{MMT}$ to represent transition rate from IDU to non-IDU who are not on MMT and who are on MMT, respectively. Drug users who are not on MMT are assumed to participate in MMT with an entry rate of γ, and $\gamma_{NIDU}$ and $\gamma_{IDU}$ represent the entry rates of non-IDU and IDU, respectively. Similarly, $\eta_{NIDU}$ and $\eta_{IDU}$ denote the drop-out rates from MMT for non-IDU and IDU, respectively. The inflow rate of drug users is denoted by *π* and drug use quitting rate denoted by *χ*. Drug users also leave the system when they died from HIV ($\mu_{\mathrm{HIV}}$), HCV ($\mu_{\mathrm{HCV}}$), drug-related harm ($\mu_{\mathrm{HARM}}$), and natural death (background death rate μ). The population flow can be represented by the system of differential equations as follows.

1. Non-IDU not on MMT ($N_{NN}$):

$$\frac{dN_{NN}}{dt}=\pi_{NN}+\omega_{NMMT}\cdot N_{IN}+\eta_{NIDU}\cdot N_{NM}-(\varphi_{NMMT}+\gamma_{NIDU}+\chi_{NN}+\mu_{NN}+\mu_{HIVNN}+\mu_{HCVNN}+\mu_{HARMNN}){\cdot N}_{NN}$$

2. Non-IDU on MMT ($N_{NM}$):

$\frac{dN_{NM}}{dt}=\pi_{NM}+\omega_{MMT}\cdot N_{IM}+\gamma_{NIDU}\cdot N_{NN}-(\varphi_{MMT}+\eta_{NIDU}+\chi_{NM}+\mu_{NM}+\mu_{HIVNM}+\mu_{HCVNM}+\mu_{HARMNM}){\cdot N}_{NM}$

3. IDU Not on MMT ($N_{IN}$):

$\frac{dN_{IN}}{dt}=\pi_{IN}+\omega_{NMMT}\cdot N_{NN}+\eta_{IDU}\cdot N_{IM}-(\omega_{NMMT}+\gamma_{IDU}+\chi_{IN}+\mu_{IN}+\mu_{HIVIN}+\mu_{HCVIN}+\mu_{HARMIN}){\cdot N}_{IN}$

1. IDU on MMT ($N_{IM}$):

$$\frac{dN_{IM}}{dt}=\pi_{IM}+\omega_{MMT}\cdot N_{NM}+\gamma_{IDU}\cdot N_{IN}-(\omega_{MMT}+\eta_{IDU}+\chi_{IM}+\mu_{IM}+\mu_{HIVIM}+\mu_{HCVIM}+\mu_{HARMIM}){\cdot N}_{IM}$$

*Modelling HIV and HCV transmission and disease progression*

In each of these four subgroups, we model four progression stages for each of HIV and HCV infections (Figure S2): (1) susceptible; (2) infected but undiagnosed; (3) diagnosed and untreated and (4) on treatment (or post-treatment for curative HCV treatment). Combining potential co-infections, there are 16 possible HIV/HCV disease stages which are applied to each of the four subgroups we previously described. In this model, HIV and HCV are transmitted through sharing of contaminated injecting equipment, and HIV can also be sexually transmitted. There is no natural clearance for HIV infection, but 15-45% HCV infected individuals clear the infection spontaneously [[1-3](#_ENREF_1)]. People living with HIV have access to antiretroviral therapy, while only interferon-based treatments for HCV are available in resource limited settings including China. The sustained virological response (SVR) rate among HCV infected patients who are treated with interferon-gamma is about 60% [[4](#_ENREF_4)]. Chronically infected individuals can progress to liver fibrosis, cirrhosis and hepatocellular carcinoma (HCC) [[5](#_ENREF_5), [6](#_ENREF_6)]. The details of the disease progression are illustrated Figure S2. We assume that the behaviour of MMT participants at enrolment (baseline) is similar to that of drug users in the community.

The model structure is demonstrated in Figure S2 and represented by a system of differential equations (page 7-8). The four diseases states were denoted by susceptible (‘S’), undiagnosed (‘U’), diagnosis (‘D’) to treatment (‘T’), respectively, for both HIV and HCV. The parameter $\lambda_{i,type}$ represents the force-of-infection of HIV and HCV in the susceptible populations (details below). The index *i* represents the type of diseases, that is, HIV or HCV, whereas ‘*type*’ represents the route of transmission, which can be one of the three: (1) sharing of injection equipment (‘*inj*’); (2) sexual transmission from male to female (‘*mf*’) and (3) sexual transmission from female to male (‘*fm*’). Among the undiagnosed, the diagnosis rate $\delta_{imn}$ represents the diagnosis rate of HIV and/or HCV infected individuals, whereas $\tau_{imn}$ represents treatment initiation rate in diagnosed HIV and/or HCV patients. Further, $\xi_{imn}$ represents treatment drop-out rates for HIV and/or HCV treatment patients and $\theta_{imn}$ represents the clearance (both natural and treatment clearance, depends on the disease states) for HCV. In these parameters, the index *m* represents the four diseases states for HIV and *n* for HCV (Figure S2).

Our model simulates HIV transmission through both unprotected and protected sexual intercourse and through using sterilized or unsterilized needles/syringes, while HCV transmitted mainly through using sterilized or unsterilized needles/syringes. Parameters are listed in Table S2.

*HIV transmission through unprotected sex:*

The probability of HIV transmission from an HIV+ female drug user to an HIV- male drug user through sexual intercourse is:

$$\lambda_{HIV, fm}=\left\{ 1-\left( 1-\beta_{HIV,fm} \right)^{n(1-p_{c})}\cdot\left[ 1-\beta_{HIV,fm}\cdot(1-\varepsilon_{c}) \right]^{np_{c}} \right\}\cdot{Prev}_{HIV, f}$$

Similarly, the probability of HIV transmission from an HIV+ male drug user to an HIV- female drug user through sexual intercourse is:

$$\lambda_{HIV, mf}=\left\{ 1-\left( 1-\beta_{HIV,mf} \right)^{n(1-p_{c})}\cdot\left[ 1-\beta_{HIV,mf}\cdot(1-\varepsilon_{c}) \right]^{np_{c}} \right\}\cdot{Prev}_{HIV,m}$$

In these equations, $\beta_{HIV,fm}$ and $\beta_{HIV,fm}$ represent per-act female-to-male and male-to-female HIV infectivity per unprotected sexual act, respectively, $n$ represent the number of sexual acts of female and male drug users per year, $p_{c}$ is the proportion of condom use in the last sexual act and $\varepsilon_{c}$ is the effectiveness of the condom. ${Prev}_{HIV,f}$ and ${Prev}_{HIV},m$ denotes the real-time prevalence of HIV of female and male drug users over time, respectively.

*HIV and HCV transmission through sharing of injection equipment*

The force-of-infection of HIV transmission through unsterilized/sterilized syringes is:

$$\lambda_{HIV, inj}=(1-\left( 1-\beta_{HIV,inj} \right)^{t_{inj}.p_{s}.\left( 1-p_{clean} \right)}.\left( 1-\beta_{HIV,inj} .\left( 1-\varphi_{clean} \right) \right)^{t_{inj}.p_{s}.p_{clean}}). {Prev}_{HIV, IDU}$$

In this equation, $\beta_{HIV,inj}$ represents HIV infectivity per sharing act among non-IDU/IDU, $t_{inj}$ represents the average times of injecting acts of each IDU per year, $p_{s}$ is the proportion of IDU who share syringes, $p_{clean}$ is the proportion of cleaned syringes; $\varphi_{clean}$ is the effectiveness of syringe cleaning. ${Prev}_{HIV, IDU}$ denotes the real-time prevalence of HIV of injecting drug users.

For IDUs, the overall force-of-infection for HIV transmission is one minus the likelihood that HIV was not transmitted even though an individual participates in a risk injection sharing and/or unprotected sex episode. It can be denoted by the expression $\lambda_{HIV}= 1-(1-\lambda_{HIV, inj}).\left[ (1-{(P_{m}\lambda}_{HIV, mf}+{P_{f}\lambda}_{HIV, fm}) \right]$, whereas $P_{m}$ and $P_{f}$ are the proportion of male and female drug users, respectively. On the contrary, the force-of-infection for HIV among non-IDUs is $\lambda_{HIV}= {P_{m}\lambda}_{HIV, mf}+{P_{f}\lambda}_{HIV, fm}$.

The force-of-infection for HCV transmission is identical to that of HIV, apart from that the per-act transmission probability of HCV is represented by $\beta_{HCV,inj}$ rather than $\beta_{HIV,inj}$.

$$\frac{dP_{SS}}{dt}=\theta_{HCVSU}\cdot P_{SU}+\theta_{HCVSD}\cdot P_{SD}+\theta_{HCVST}\cdot P_{ST}-(\lambda_{HIVSS}+\lambda_{HCVSS})\cdot P_{SS}$$

$$\frac{dP_{SU}}{dt}=\lambda_{HCVSS}\cdot P_{SS}-(\lambda_{HIVSU}+\delta_{HCVSU}+\theta_{HCVSU})\cdot P_{SU}$$

$$\frac{dP_{SD}}{dt}=\delta_{HCVSU}\cdot P_{SU}-(\lambda_{HIVSD}+\tau_{HCVSD}+\theta_{HCVSD})\cdot P_{SD}$$

$$\frac{dP_{ST}}{dt}=\tau_{HCVSD}\cdot P_{SD}-(\lambda_{HIVST}+\theta_{HCVST})\cdot P_{ST}$$

$$\frac{dP_{US}}{dt}=\lambda_{HIVSS}\cdot P_{SS}+\theta_{HCVUU}\cdot P_{UU}+\theta_{HCVUD}\cdot P_{UD}+\theta_{HCVUT}\cdot P_{UT}-(\lambda_{HCVUS}+\delta_{HIVUS})\cdot P_{US}$$

$$\frac{dP_{UU}}{dt}=\lambda_{HCVUS}\cdot P_{US}+\lambda_{HIVSU}\cdot P_{SU}-(\delta_{HIVUU}+\delta_{HCVUU}+\theta_{HCVUU})\cdot P_{UU}$$

$$\frac{dP_{UD}}{dt}=\delta_{HCVUU}\cdot P_{UU}+\lambda_{HIVSD}\cdot P_{SD}-(\delta_{HIVUD}+\tau_{HCVUD}+\theta_{HCVUD})\cdot P_{UD}$$

$$\frac{dP_{UT}}{dt}=\tau_{HCVUD}\cdot P_{UD}+\lambda_{HIVST}\cdot P_{ST}-(\delta_{HIVUT}+\theta_{HCVUT})\cdot P_{UT}$$

$$\frac{dP_{DS}}{dt}=\delta_{HIVUS}\cdot P_{US}+\theta_{HCVDU}\cdot P_{DU}+\theta_{HCVDD}\cdot P_{DD}+\theta_{HCVDT}\cdot P_{DT}+\xi_{HIVTS}\cdot P_{TS}-(\lambda_{HCVDS}+\tau_{HIVDS})\cdot P_{DS}$$

$$\frac{dP_{DU}}{dt}=\delta_{HIVUU}\cdot P_{UU}+\lambda_{HCVDS}\cdot P_{DS}+\xi_{HIVTU}\cdot P_{TU}-({\delta_{HCVDU}+\tau}_{HIVDU}+\theta_{HCVDU})\cdot P_{DU}$$

$$\frac{dP_{DD}}{dt}=\delta_{HCVDU}\cdot P_{DU}+\delta_{HIVUD}\cdot P_{UD}+\xi_{HIVTD}\cdot P_{TD}-({\tau_{HIVDD}+\tau}_{HCVDD}+\theta_{HCVDD})\cdot P_{DD}$$

$$\frac{dP_{DT}}{dt}=\tau_{HCVDD}\cdot P_{DD}+\delta_{HIVUT}\cdot P_{UT}+\xi_{HIVTT}\cdot P_{TT}-(\tau_{HIVDT}+\theta_{HCVDT})\cdot P_{DT}$$

$$\frac{dP_{TS}}{dt}=\tau_{HIVDS}\cdot P_{DS}+\theta_{HCVTU}\cdot P_{TU}+\theta_{HCVTD}\cdot P_{TD}+\theta_{HCVTT}\cdot P_{TT}-(\lambda_{HCVTS}+\xi_{HIVTS})\cdot P_{TS}$$

$$\frac{dP_{TU}}{dt}=\lambda_{HCVTS}\cdot P_{TS}+\tau_{HIVDU}\cdot P_{DU}-({\xi_{HIVTU}+\delta}_{HCVTU}+\theta_{HIVTU})\cdot P_{TU}$$

$$\frac{dP_{TD}}{dt}=\delta_{HCVTU}\cdot P_{TU}+\tau_{HIVDD}\cdot P_{DD}-({\xi_{HIVTD}+\theta}_{HCVTD}+\tau_{HCVTD})\cdot P_{TD}$$

$$\frac{dP_{TT}}{dt}=\tau_{HCVTD}\cdot P_{TD}+\tau_{HIVDT}\cdot P_{DT}-({\xi_{HIVTT}+\theta}_{HCVTT})\cdot P_{TT}$$

***Model calibration***

We calibrated the model to available epidemiological data. We used a Latin Hypercube sampling method to sample collected epidemiological (Table S1) and behavioural (Table S2) indicators within their uncertainty bounds. This was repeated 10,000 times. For each simulation, we estimated the goodness of fit between the output results and observed data based on the standard deviation of errors. Simulated outputs were ranked according to their goodness of fit, and the highest ranked 5% was regarded as the calibrated model estimates to the epidemic trend and used to generate the model outputs with 95% confidence intervals (CIs).

***Effectiveness and cost-effectiveness evaluation***

The model generated epidemiological indicators for HIV and HCV in both drug users (DUs) and injecting drug users (IDUs), and predicted these indicators in the absence of MMT in counterfactual scenarios. These include prevalence, incidence, the estimated number of new infections and diagnoses, the number of individuals on treatment and deaths. We calculated the effectiveness of MMT in China as the differences in these indicators by comparing MMT and ‘no MMT’ scenarios. We hence obtained the number of HIV and HCV cases averted, drug use person-years averted, HIV and HCV related deaths averted, and harm-related deaths averted as a result of MMT. The number of HIV-, HCV- and harm-related disability adjusted life years (DALYs, Table S4) averted were calculated based on simulated results and health utilities in each disease stage among drug users in China. We estimated the total MMT investment over the past decade and calculated the amount for each DALY averted by reducing HIV, HCV and harm in the Chinese drug users, respectively. The unit cost for drug use and HIV/HCV treatment was listed in Table S5. We used 3.5% discounting rate in our economic analysis.

***Uncertainty analysis***

Uncertainty analysis was used to identify the variations in indicators and inform the accuracy of output results, by describing the distribution, central and discrete tendency of the outputs. We used descriptive statistics to describe the distribution and uncertainties of model outputs.

# Figure S1. Model structure for the exchanges between subgroups of drug users

# Figure S2. Disease progression of HIV and HCV infections in each subgroup of drug users.

**Table S1 Population size and HIV and HCV prevalence in drug users and IDUs in China during 2004-2015**

| **Description** |  |  |  |  |  | **Year** |  |  |  |  |  |  | **Reference** |
| --- | --- | --- | --- | --- | --- | --- | --- | --- | --- | --- | --- | --- | --- |
|  | **2004** | **2005** | **2006** | **2007** | **2008** | **2009** | **2010** | **2011** | **2012** | **2013** | **2014** | **2015** |
| **Population size** |  |  |  |  |  |  |  |  |  |  |  |  |  |
| The number of registered drug users in China (million) | 0.75 | 0.79 | NA | 0.96 | 1.13 | 1.34 | 1.55 | 1.79 | 2.10 | 2.48 | 2.96 | 3.49 | [[7-18](#_ENREF_7)] |
| The number of drug users in methadone maintenance treatment in China (thousand) | 2.97 | 4.14 | 37.00 | 58.00 | 93.70 | 110.00 | 130.00 | 134.00 | 208.00 | 201.73 | 187.00 | 208.00 | [[7-18](#_ENREF_7)] |
| The number of drug users on antiretroviral therapy in China (thousand) | 4.82 | 7.21 | 10.02 | 12.19 | 14.66 | NA | 26.01 | 35.91 | 42.25 | 47.04 | NA | NA | [[19-27](#_ENREF_19)] |
| The number of diagnosed HIV drug users in China (thousand) | 21.04 | 17.99 | 15.03 | 13.18 | 16.76 | 17.20 | 15.75 | 14.67 | 7.69 | 6.50 | 5.78 | 4.68 | [[28-35](#_ENREF_28)] |
| Accumulated number of injection drug users diagnosed with HIV in China (thousand) | 36.79 | 55.34 | NA | 86.05 | 90.72 | 103.00 | 113.66 | 126.30 | 133.99 | 140.48 | 146.26 | 150.94 | [[7](#_ENREF_7), [8](#_ENREF_8), [19](#_ENREF_19), [21](#_ENREF_21), [22](#_ENREF_22), [31-36](#_ENREF_31)] |
| **HIV and HCV prevalence** |  |  |  |  |  |  |  |  |  |  |  |  |  |
| HIV prevalence among the general Chinese population (%) | 0.07 (constant) | | | | | | | | | | | | [[25](#_ENREF_25)] |
| HIV prevalence among drug users in China (%) | 6.33 | 7.47 | 7.00 | 6.92 | 5.00 | 6.11 | 4.44 | 4.00 | 3.70 | 3.87 | 3.44 | 3.30 | [[22](#_ENREF_22), [37](#_ENREF_37), [38](#_ENREF_38)] |
| HIV prevalence among injection drug users in China (%) | 12.58 | 10.93 | 7.18 | 7.64 | 8.33 | 8.00 | 6.95 | 6.43 | 6.30 | 6.31 | 6.00 | NA | [[37](#_ENREF_37), [39](#_ENREF_39), [40](#_ENREF_40)] |
| HCV prevalence among drug users in China (%) | NA | NA | NA | NA | 45.00 | 40.10 | 41.81 | 42.82 | 41.55 | NA | NA | NA | [[41-43](#_ENREF_41)] |
| HCV prevalence among injection drug users in China (%) | NA | NA | NA | NA | 61.40 | 55.30 | 61.00 | 63.80 | 63.20 | NA | NA | NA | [[41](#_ENREF_41), [44](#_ENREF_44)] |

**Table S2. Risk injecting and sexual behaviours in drug users, HIV and HCV infectivity, diagnosis and treatment rates in each drug user subgroups.**

| **Parameter** | **Sampled range** | | | | | | **Reference** |
| --- | --- | --- | --- | --- | --- | --- | --- |
|  | **Not on MMT** | | **On MMT** | | | |  |
|  | **Non-IDU** | **IDU** | **Non-IDU** | | | **IDU** |  |
| **Injecting behaviours of drug users** | | | | | | | |
| Average injecting frequency of IDU each year | —— | 387.3 (6-1440) | 66.5-95.0 (Time-dependent)*^a^* | | | | [[45](#_ENREF_45), [46](#_ENREF_46)] |
| Proportion of IDU who share syringe | —— | 41% (30-52)%^*^ | 12-29% (Time-dependent)*^b^* | | | | [[46-60](#_ENREF_46)] |
| Proportion of cleaned syringes | —— | 51% (33-68)% ^*^ | —— | | | 51% (33-68)% ^*^ | [[56](#_ENREF_56), [60](#_ENREF_60)] |
| Effectiveness of syringe cleaning | —— | 86% (84-89)% | —— | | | 86% (84-89)% | [[61-63](#_ENREF_61)] |
| Proportion of who quit drug use each year | 2% (1.5-2.5)% | | | | | | [[64](#_ENREF_64), [65](#_ENREF_65)] |
| Proportion of drop-out of Chinese MMT participants | —— | —— | 46.5% (41-53)%^*^ | | | 47.5% (41-53)%^*^ | [[66-69](#_ENREF_66)]*^c^* |
| Proportion of conversion from non-IDU to IDU | 33.2% (13.8-52.7)% ^*^ | —— | 33.2% (13.8-52.7)%^*^ | | | —— | [[70-72](#_ENREF_70)] |
| Proportion of conversion from IDU to non-IDU | —— | 1.0% (0.8-1.2)% | —— | | | 1.0% (0.8-1.2)% | Model assumption |
|  | | | | | | | |
| **Sexual behaviours of drug users** | | | | | | | |
| The number of sexual acts of drug users per year | 56 (36-75) | | | | | | [[73](#_ENREF_73)] |
| Proportion of consistent condom use in the past three months | 26.0 (21.9-30.6)%^♂^ | | 22.5-26.4%  (Time-dependent) *^d^* | | | | [[66](#_ENREF_66), [73](#_ENREF_73)] |
| Percentage of female among drug users | 16.1% (13.2-19.0%)^*^ | | 15.7% (12.2-19.3%)^*^ | | | | [[44](#_ENREF_44), [66](#_ENREF_66), [74](#_ENREF_74)] |
|  | | | | | | | |
| **HIV transmission, screening and treatment** | | | | | | | |
| HIV infectivity per sharing act among DU | —— | 0.23% (0-2.38)% | | —— | 0.23% (0-2.38)% | | [[75](#_ENREF_75)] |
| HIV infectivity from female to male per unprotected sexual act among DU | 0.04% (0.01-0.14)% | | | | | | [[76](#_ENREF_76)] |
| HIV infectivity from male to female per unprotected sexual act among DU | 0.08% (0.06-0.11)% | | | | | | [[76](#_ENREF_76)] |
| Proportion of screened HIV patients | 40.7% (38.1-43.3)% | | 95% (90-99)% | | | | [[25](#_ENREF_25), [77](#_ENREF_77), [78](#_ENREF_78)] |
| ART initiation rate in diagnosed HIV+ drug users | 15-60 %  (Time-dependent) *^e^* | | 42-85%  (Time-dependent) *^f^* | | | | [[25](#_ENREF_25), [79](#_ENREF_79)] |
| ART drop-out rate | 37.2 (29.6-44.7)%^*^ | | 17.3% (16.3-18.3)%^*^ | | | | [[80-85](#_ENREF_80)] |
|  | | | | | | | |
| **HCV transmission, screening and treatment** | | | | | | | |
| Infectivity of HCV per sharing act in DU | —— | 1.8% (1.5-4.0)% | | —— | 1.8% (1.5-4.0)% | | [[86-96](#_ENREF_86)]*^g^* |
| Annual HCV screening uptake (%) |  | |  | | | |  |
| *HIV- drug users* | 28.0% (23.4-32.6%) | | 78.4% (77.6-79.2)% | | | | [[77](#_ENREF_77), [97](#_ENREF_97)] |
| *HIV+ drug users* | 52.2% (40.2-64.2%) | | 78.4% (77.6-79.2)% | | | | [[77](#_ENREF_77), [98](#_ENREF_98)] |
| Proportion of HCV-infected drug users initiate IFN-α treatment every year | 38.8% (28.1-49.5)%^*^ | | | | | | [[99](#_ENREF_99)] |
| HCV clearance |  | | | | | |  |
| *Natural clearance rate* | 15.4% (11.5-19.3)% | | | | | |  |
| *Treatment clearance rate* | 67.0% (56.4-77.7)%^*^ | | | | | | [[100-106](#_ENREF_100)] |
|  | | | | | | | |
| **Risk of death** | | | | | | | |
| Natural death (background death) | 0.7% (0.5-0.9%) | | | | | | [[107](#_ENREF_107)] |
| Drug overdose | 0.74 (0.2-1.0)% | 0.84 (0.3-1.2)% | 0.64% (0.2-0.9)% | | | 0.73% (0.2-1.0)% | [[108](#_ENREF_108)]*^h^* |
| Undiagnosed HIV infection | 12.5% (9.4-15.6)% *^i^* | | | | | | [[109](#_ENREF_109)] |
| Diagnosed AIDS patients | 11.8% (10.7-12.9)%^*^ | | | | | | [[110](#_ENREF_110), [111](#_ENREF_111)] |
| AIDS patients on ART | 2.5% (2.3-2.8%)^*^ | | 1.4% (1.4-1.5)% | | | | [[81](#_ENREF_81), [110](#_ENREF_110), [112-114](#_ENREF_112)] |
| Undiagnosed HCV infection | 0.2% (0.05-0.35)%^*^ | | | | | | [[115](#_ENREF_115), [116](#_ENREF_116)] |
| Infected HCV individuals on IFN-α treatment | 0.11% (0.03-0.19%) | | | | | | [[115-117](#_ENREF_115)]*^j^* |
|  |  | | | | | |  |
| **Notes for time-dependent risk behaviours:** In this model, we have obtained strong evidence that drug users on MMT changed their drug use and injecting behaviours. With the expansion of MMT over time, more drug users experienced behavioural changes, and we observed an overall declining trend of risk behaviours overtime. With the collected information on the number of new MMT entrants, the number of people on MMT and adherence rate in each year, we calculated the distribution of treatment duration in MMT clients in each year accordingly. Based on which, we estimated an overall weighted average of risk behaviours for each calendar year (below figure). In contrast, we did not identify any strong evidence that suggests changes in risk behaviours in drug users (including PWID) outside MMT. We, therefore, assume their behaviours remain unchanged over time. | | | | | | | |
| 1. Average injecting frequency of IDU on MMT each year | | | | | | | |
| 1. Proportion of IDU who share syringe during MMT | | | | | | | |
| 1. According to literature, the odds to retain in MMT was 1.02 in injecting drug users compared with non-injecting drug users [71]. The retention rate of MMT participants was 47% (41-53)% by meta-analysis. Thus, we calculated the retention rate for injecting drug users in MMT [47.5% (41-53)%] and non-injecting drug users in MMT [46.5% (41-53)%]. | | | | | | | |
| 1. Proportion of consistent condom use in the past three months in drug users on MMT | | | | | | | |
| 1. ART initiation rate in diagnosed HIV+ drug users outside MMT | | | | | | | |
| 1. ART initiation rate in diagnosed HIV+ drug users on MMT | | | | | | | |
| 1. No empirical studies reported the infectivity of HCV per sharing act by DUs. We used the previously estimate by Jisoo A. Kwon et al. [[86](#_ENREF_86)]. This study estimated the infectivity of HCV per sharing act by summarizing the numbers on the transmission risk of HCV in an occupational setting due to needle stick injury [[87-94](#_ENREF_87)]. The estimation has been used in many following mathematical modelling studies [[95](#_ENREF_95), [96](#_ENREF_96)]. | | | | | | | |
| 1. The mortality due to overdose was 0.736% (52/7069) based on previous literature[[108](#_ENREF_108)]. Injecting drug users had 1.15 times higher mortality, while non-MMT drug users had 1.14 times higher mortality. We thus estimated the mortality due to overdose was 0.74 (0.2-1.0)% for non-injecting drug users, 0.84 (0.3-1.2)% for injecting drug users, 0.64% (0.2-0.9)% for non-injecting drug users in MMT and 0.73% (0.2-1.0)% for injecting drug users in MMT. | | | | | | | |
| 1. AIDS patients were diagnosed when symptoms occur. Based on the literature, the median time of latent HIV was eight years among HIV positive drug users in China. We, therefore, estimated the 1/8 (12.5%) of the patients were undiagnosed. | | | | | | | |
| 1. For drug users who receive INF-η treatment for HCV, the sustained virologic response for the antiviral therapy is 45% [[117](#_ENREF_117)]. We, therefore, assume the death rate is reduced by 45% as a result of the treatment. | | | | | | | |

^♂^Data were consistent with figure 2

*data were estimated using the meta-analysis method

**Table S3. Disability-adjusted-life-years (DALYs) weights in each drug-use, HIV and HCV infection stages.**

| **Description** | **DALY Weight** | **Notes/Reference** |
| --- | --- | --- |
| **DALY weight due to HIV infection** |  |  |
| HIV susceptible | 0 |  |
| Undiagnosed HIV positive | 0.012 | Footnote *a*, [[118](#_ENREF_118)] |
| Diagnosed HIV/AIDS without receiving ART | 0.582 | Footnote *b*, [[119](#_ENREF_119)] |
| On ART | 0.078 | [[119](#_ENREF_119)] |
| **DALY weight due to HCV infection** |  |  |
| HCV susceptible | 0 | Footnote *c* |
| Undiagnosed HCV positive | 0.038 | Footnote *d*, [[120](#_ENREF_120)] (supplementary materials pp 6) |
| Diagnosed HCV positive | 0.111 | Footnote *e*, [[120](#_ENREF_120)] (supplementary materials pp 6) |
| On HCV treatment | 0.006 | Footnote *f*, [[120](#_ENREF_120)] (supplementary materials pp 6) |
| **DALY weight due to drug use**^d^ |  |  |
| Injecting drug users who were not on MMT | 0.652 | Footnote *g* |
| Non-injecting drug users who were not on MMT | 0.544 |
| Injecting drug users who were on MMT | 0.372 | Footnote *h* |
| Non-injecting drug users who were on MMT | 0.310 |

**Footnote:**

1. Undiagnosed HIV+ individuals were assumed to be in the early chronic stage of HIV infection without symptoms, who have a DALY weight of 0.012 (0.006-0.023).
2. Diagnosed HIV+ individuals were assumed in a late chronic stage with symptoms of AIDS, who have a DALY weight of 0.582 (0.406-0.743).
3. We assumed HCV susceptible individuals are similar to health individuals and hence have a DALY weight of 0.
4. We assumed undiagnosed HCV+ individuals are at a stage between ‘infectious diseases, acute episode, mild’ (disability weight, 0.0006 [0.002-0.012]) and ‘’infectious disease, acute episode, moderate’ (disability weight, 0.0051 [0.032-0.074]). We hence used the lower 95% confidence interval of the mild stage and the upper 95% confidence interval of the moderate stage. We estimate the mean value to be average of these confidence intervals; hence, the estimated DALY weight is 0.038 (0.002-0.074).
5. We assumed diagnosed HCV+ individuals are at a stage between ‘infectious diseases, acute episode, moderate’ (disability weight, 0.0051 [0.032-0.074]). and ‘infectious disease, acute episode, severe’ (disability weight, 0.133 [0.088-0.19]). We hence used the lower 95% confidence interval of the mild stage and the upper 95% confidence interval of the moderate stage. We estimate the mean value to be average of these confidence intervals; hence the estimated DALY weight is 0.111 (0.032-0.19).
6. We assumed individuals who on HCV treatment but yet achieve a total clearance were similar to individuals at a stage of “infectious disease, mild” (DALYs weight 0.006 (0.002-0.012).
7. The calculation of DALY weight of non-IDUs and IDU at MMT enrolment are based on three pieces of information: (1) Shi et al., [[121](#_ENREF_121)] reported that 1%, 34.3% and 64.7% drug users had mild (DALYs: 0.335), moderate (DALYs: 0.516) and severe (0.697) opioid dependence at MMT enrolment; this led to an overall weighted average of 0.631 of all MMT starters, including both non-IDUs and IDUs; (2) Our literature review suggested that 80% of MMT starters are IDUs at enrolment; (3) Barrio et al., [[122](#_ENREF_122)] reported that Severity of Dependence Scale (SDS) score in IDUs are 20% higher than that of non-IDUs, we,, assume that DALY weight of drug dependence is also 20% higher in IDUs than non-IDUs. These conditions result in solutions for DALY weights for IDUs and non-IDUs at MMT enrolment to be 0.652 and 0.544, respectively. We use these values as proxy for DALY weights in IDUs and non-IDUs who are not on MMT.
8. Shi et al., [[121](#_ENREF_121)] reported that the severity of opioid dependence in drug users who have participated in MMT for six months (SDS=6.3) was 43% lower than that of drug users at MMT enrolment (SDS=11.1). We, therefore, assumed the DALY weights due to drug dependence are also reduced by the same proportion. This leads to the DALY weights calculated for IDUs and non-IDUs at MMT to be 0.309 and 0.370, respectively.

**Table S4. Costing parameters for drug use and HIV/HCV treatment**

| **Description** | **Cost (dollars)** | **Reference** |
| --- | --- | --- |
| **Cost spent on illicit drugs yearly** |  |  |
| Non-injection drug users who were not on MMT | 2000 | [[123-126](#_ENREF_123)] |
| Injection drug users who were not on MMT | 7000 |
| Non-injection drug users who were on MMT | 600 |
| Injection drug users who were on MMT | 600 |
| **Cost spent on HIV treatment yearly** |  |  |
| HIV susceptible drug users | 3 | [[127-130](#_ENREF_127)] |
| Undiagnosed HIV+ drug users | 500 |
| Diagnosed HIV+ drug users | 567.7 |
| Drug users who were on ART | 4000 | [[128-131](#_ENREF_128)] |
| **Cost spent on HCV treatment yearly** |  |  |
| HCV susceptible drug users | 1.5 | [[127](#_ENREF_127), [129](#_ENREF_129), [132](#_ENREF_132)] |
| Undiagnosed HCV+ drug users | 879 |
| Diagnosed HCV+ drug users | 879 |
| Drug users who were on HCV treatment | 6000 | [[127](#_ENREF_127), [129](#_ENREF_129), [132](#_ENREF_132), [133](#_ENREF_133)] |

**Table S5. Costing parameters for MMT programme**

| **Category** | **Item** | **Description** | **Cost*** | | | **Reference** |
| --- | --- | --- | --- | --- | --- | --- |
| Personal cost (per person) | Provision of methadone | Methadone | $0.12 | person | day | [[127](#_ENREF_127)] |
|  |  | Paper cups | $0.02 | person | time | [[127](#_ENREF_127)] |
|  | Urine for drug usage | Cost of materials includinng the test paper, reagents, cups etc. | $0.49 | person | time | [[127](#_ENREF_127)] |
| Clinic cost (per clinic) | Advertisement materials | Cost of publishing advertisement materials | $809.06 | clinic | year | [[127](#_ENREF_127)] |
|  | Personnel training | Cost for personnel training on MMT | $1,972.8 | clinic | year | [[134](#_ENREF_134)] |
|  | Salary | Salaries, wages and others | $145846.9 | clinic | year | [[134](#_ENREF_134)] |
|  | Building operation and maintenance | Cost of electricity, water, heating, fuel, telephone, telex, insurance, cleaning, painting, repairs ect. | $7567.2 | clinic | year | [[134](#_ENREF_134)] |
|  | Vehicle operation and maintenance | Petrol, diesel, lubricants, tyres, Spare parts, registration, insurance | $833.3 | clinic | year | [[134](#_ENREF_134)] |

*The exchange rate is 6.18. Annual cost of the Chinese MMT programme were calculated using annual number of MMT clinics multiply the sum of all clinic costs (advertisement materials, personnel training, salary, building operation and maintenance, and vehicle operation and maintenance), then add the cost of total personal costs, which were calculated using annual number of MMT participants multiply the sum of all personal costs (provision of methadone, urine for drug usage).

**Table S6. CHEERS checklist — Items to include when reporting economic evaluations of health interventions**

| **Section/item** | **Item No.** | **Recommendation** | **Reported on Page No./ line No.** |
| --- | --- | --- | --- |
|  |  |  |  |
| **Title and abstract** | | |  |
| Title | 1 | Identify the study as an economic evaluation or use more specific terms such as “cos-effectiveness analysis”, and describe the interventions compared. | Page 1,line 1 -2 |
| Abstract | 2 | Provide a structured summary of objectives, perspective, setting, methods | Page 2-3 |
|  |  | (including study design and inputs), results (including base case and uncertainty analyses), and conclusions. |  |
| **Introduction** | |  |  |
| Background and objectives | 3 | Provide an explicit statement of the broader context for the study. | Page 4-5 |
|  |  | Present the study question and its relevance for health policy or practice decisions. | Page 5 |
| **Methods** |  |  |  |
| Target population and subgroups | 4 | Describe the characteristics of the base case population and subgroups analysed, including why they were chosen. | Page 7, line 8-13 |
| Setting and location | 5 | State relevant aspects of the system(s) in which the decision(s) need(s) to be made. | Page 7, line 8 |
| Study perspective | 6 | Describe the perspective of the study and relate this to the costs being evaluated. | Page 5, line 19-21/ Supplementary material Table S3-5 |
| Comparators | 7 | Describe the interventions or strategies being compared and state why they were chosen. | Page 8, line 8-10 |
| Time horizon | 8 | State the time horizon(s) over which costs and consequences are being evaluated and say why appropriate. | Page 8, line 8-10 |
| Discount rate | 9 | Report the choice of discount rate(s) used for costs and outcomes and say why appropriate. | Page 8, line 20-22 |
| Choice of health outcomes | 10 | Describe what outcomes were used as the measure(s) of benefit in the evaluation and their relevance for the type of analysis performed. | Page 8, line 10-20 |
| Measurement of effectiveness | 11a | Single study-based estimates: Describe fully the design features of the single effectiveness study and why the single study was a sufficient source of clinical effectiveness data. | NA |
| Measurement and valuation of preference based outcomes | 12 | If applicable, describe the population and methods used to elicit preferences for outcomes. | Page 8, line 8-20 |
| Estimating resources and costs | 13a | Single study-based economic evaluation: Describe approaches used to estimate resource use associated with the alternative interventions. Describe primary or secondary research methods for valuing each resource item in terms of its unit cost. Describe any adjustments made to approximate to opportunity costs. | NA |
| Currency, price date, and conversion | 14 | Report the dates of the estimated resource quantities and unit costs. Describe methods for adjusting estimated unit costs to the year of reported costs if necessary. Describe methods for converting costs into a common currency base and the exchange rate. | Page 8, line 21-22 |
| Choice of model | 15 | Describe and give reasons for the specific type of decision-analytical model used. Providing a figure to show model structure is strongly recommended. | Supplementary materials, Figure S1-S2 |
| Assumptions | 16 | Describe all structural or other assumptions underpinning the decision-analytical model. | Page 7, line 8-25 |
| Analytical methods | 17 | Describe all analytical methods supporting the evaluation. This could include methods for dealing with skewed, missing, or censored data; extrapolation methods; methods for pooling data; approaches to validate or make adjustments (such as half cycle corrections) to a model; and methods for handling population heterogeneity and uncertainty. | Page 8, line 1-6 |
| **Results** |  |  |  |
| Study parameters | 18 | Report the values, ranges, references, and, if used, probability distributions for all parameters. Report reasons or sources for distributions used to represent uncertainty where appropriate. Providing a table to show the input values is strongly recommended. | Supplementary materials |
| Incremental costs and outcomes | 19 | For each intervention, report mean values for the main categories of estimated costs and outcomes of interest, as well as mean differences between the comparator groups. If applicable, report incremental cost-effectiveness ratios. | Page 12, Table 1 |
| Characterising uncertainty | 20a | Single study-based economic evaluation: Describe the effects of sampling uncertainty for the estimated incremental cost and incremental effectiveness parameters, together with the impact of methodological assumptions (such as discount rate, study perspective). | NA |
|  | 20b | Model-based economic evaluation: Describe the effects on the results of uncertainty for all input parameters, and uncertainty related to the structure of the model and assumptions. | Page 12, Table 1 |
| Characterising heterogeneity | 21 | If applicable, report differences in costs, outcomes, or cost-effectiveness that can be explained by variations between subgroups of patients with different baseline characteristics or other observed variability in effects that are not reducible by more information. | NA |
| **Discussion** | |  |  |
| Study findings, limitations, generalisability, and current knowledge | 22 | Summarise key study findings and describe how they support the conclusions reached. Discuss limitations and the generalisability of the findings and how the findings fit with current knowledge. | Page 14-16 |
| Other |  |  |  |
| Source of funding | 23 | Describe how the study was funded and the role of the funder in the identification, design, conduct, and reporting of the analysis. Describe other nonmonetary sources of support. | Page 18 |
| Conflicts of interest | 24 | Describe any potential for conflict of interest of study contributors in accordance with journal policy. In the absence of a journal policy, were commend authors | Conflicts of interest statement |
|  |  | comply with International Committee of Medical Journal Editors recommendations. |  |

| **Section/topic** | **#** | **Checklist item** | **Reported on page #** |
| --- | --- | --- | --- |
| **TITLE** | | |  |
| Title | 1 | Identify the report as a systematic review, meta-analysis, or both. | NA |
| **ABSTRACT** | | |  |
| Structured summary | 2 | Provide a structured summary including, as applicable: background; objectives; data sources; study eligibility criteria, participants, and interventions; study appraisal and synthesis methods; results; limitations; conclusions and implications of key findings; systematic review registration number. | Abstract |
| **INTRODUCTION** | | |  |
| Rationale | 3 | Describe the rationale for the review in the context of what is already known. | Introduction paragraph 4 |
| Objectives | 4 | Provide an explicit statement of questions being addressed with reference to participants, interventions, comparisons, outcomes, and study design (PICOS). | NA |
| **METHODS** | | |  |
| Protocol and registration | 5 | Indicate if a review protocol exists, if and where it can be accessed (e.g., Web address), and, if available, provide registration information including registration number. | NA |
| Eligibility criteria | 6 | Specify study characteristics (e.g., PICOS, length of follow-up) and report characteristics (e.g., years considered, language, publication status) used as criteria for eligibility, giving rationale. | Material and Method, Literature search  Supplementary Material, Selection criteria |
| Information sources | 7 | Describe all information sources (e.g., databases with dates of coverage, contact with study authors to identify additional studies) in the search and date last searched. | Material and Method, Literature search  Supplementary Material, literature search, paragraph 2 |
| Search | 8 | Present full electronic search strategy for at least one database, including any limits used, such that it could be repeated. | Material and Method, Literature search  Supplementary Material, Search strategies |
| Study selection | 9 | State the process for selecting studies (i.e., screening, eligibility, included in systematic review, and, if applicable, included in the meta-analysis). | Material and Method, Literature search  Supplementary Material, Selection criteria |
| Data collection process | 10 | Describe method of data extraction from reports (e.g., piloted forms, independently, in duplicate) and any processes for obtaining and confirming data from investigators. | Material and Method, Literature search  Supplementary Material, Data extraction and analysis |
| Data items | 11 | List and define all variables for which data were sought (e.g., PICOS, funding sources) and any assumptions and simplifications made. | Material and Method, Literature search  Supplementary Material, literature search, paragraph 1 |
| Risk of bias in individual studies | 12 | Describe methods used for assessing risk of bias of individual studies (including specification of whether this was done at the study or outcome level), and how this information is to be used in any data synthesis. | NA |
| Summary measures | 13 | State the principal summary measures (e.g., risk ratio, difference in means). | Material and Method, Literature search paragraph 3 |
| Synthesis of results | 14 | Describe the methods of handling data and combining results of studies, if done, including measures of consistency (e.g., I^2^) for each meta-analysis. | Material and Method, Literature search paragraph 3 |

Page 1 of 2

| **Section/topic** | **#** | **Checklist item** | **Reported on page #** |
| --- | --- | --- | --- |
| Risk of bias across studies | 15 | Specify any assessment of risk of bias that may affect the cumulative evidence (e.g., publication bias, selective reporting within studies). | NA |
| Additional analyses | 16 | Describe methods of additional analyses (e.g., sensitivity or subgroup analyses, meta-regression), if done, indicating which were pre-specified. | NA |
| **RESULTS** | | |  |
| Study selection | 17 | Give numbers of studies screened, assessed for eligibility, and included in the review, with reasons for exclusions at each stage, ideally with a flow diagram. | Results, MMT Effectively Reduced Risk Behaviours of DUs |
| Study characteristics | 18 | For each study, present characteristics for which data were extracted (e.g., study size, PICOS, follow-up period) and provide the citations. | NA |
| Risk of bias within studies | 19 | Present data on risk of bias of each study and, if available, any outcome level assessment (see item 12). | NA |
| Results of individual studies | 20 | For all outcomes considered (benefits or harms), present, for each study: (a) simple summary data for each intervention group (b) effect estimates and confidence intervals, ideally with a forest plot. | Results, MMT Effectively Reduced Risk Behaviours of DUs |
| Synthesis of results | 21 | Present results of each meta-analysis done, including confidence intervals and measures of consistency. | Effectively Reduced Risk Behaviours of DUs |
| Risk of bias across studies | 22 | Present results of any assessment of risk of bias across studies (see Item 15). | NA |
| Additional analysis | 23 | Give results of additional analyses, if done (e.g., sensitivity or subgroup analyses, meta-regression [see Item 16]). | NA |
| **DISCUSSION** | | |  |
| Summary of evidence | 24 | Summarize the main findings including the strength of evidence for each main outcome; consider their relevance to key groups (e.g., healthcare providers, users, and policy makers). | NA |
| Limitations | 25 | Discuss limitations at study and outcome level (e.g., risk of bias), and at review-level (e.g., incomplete retrieval of identified research, reporting bias). | Discussion |
| Conclusions | 26 | Provide a general interpretation of the results in the context of other evidence, and implications for future research. | Conclusion |
| **FUNDING** | | |  |
| Funding | 27 | Describe sources of funding for the systematic review and other support (e.g., supply of data); role of funders for the systematic review. | Funding |

*From:*  Moher D, Liberati A, Tetzlaff J, Altman DG, The PRISMA Group (2009). Preferred Reporting Items for Systematic Reviews and Meta-Analyses: The PRISMA Statement. PLoS Med 6(7): e1000097. doi:10.1371/journal.pmed1000097

**Reference**:

1. Kong F, Pan Y, Chi X, Wang X, Chen L, Lv J, Sun H, Wu R, Jin J, Yu G *et al*: **Factors Associated with Spontaneous Clearance of Hepatitis C Virus in Chinese Population**. *BioMed Research International* 2014, **2014**:6.

2. Smith DJ, Jordan AE, Frank M, Hagan H: **Spontaneous viral clearance of hepatitis C virus (HCV) infection among people who inject drugs (PWID) and HIV-positive men who have sex with men (HIV+ MSM): a systematic review and meta-analysis**. *BMC Infectious Diseases* 2016, **16**(1):471.

3. Westbrook RH, Dusheiko G: **Natural history of hepatitis C**. *Journal of Hepatology*, **61**(1):S58-S68.

4. Wang FS, Fan JG, Zhang Z, Gao B, Wang HY: **The global burden of liver disease: the major impact of China**. *Hepatology (Baltimore, Md)* 2014, **60**(6):2099-2108.

5. Duan Z, Jia JD, Hou J, Lou L, Tobias H, Xu XY, Wei L, Zhuang H, Pan CQ: **Current challenges and the management of chronic hepatitis C in mainland China**. *Journal of clinical gastroenterology* 2014, **48**(8):679-686.

6. Chuang W-L, Yu M-L: **Host factors determining the efficacy of hepatitis C treatment**. *Journal of Gastroenterology* 2013, **48**(1):22-30.

7. Security MoP: **Annual Report on Drug Control in China, 2005**. In: *Annual Report on Drug Control in China.* Beijing: Ministry of Public Security; 2005: 20.

8. Security MoP: **Annual Report on Drug Control in China, 2006**. In: *Annual Report on Drug Control in China.* Beijing: Ministry of Public Security; 2006: 20.

9. Security MoP: **Annual Report on Drug Control in China, 2007**. In: *Annual Report on Drug Control in China.* Beijing: Ministry of Public Security; 2007: 20.

10. Security MoP: **Annual Report on Drug Control in China,2008**. In: *Annual Report on Drug Control in China.* Beijing: Ministry of Public Security; 2008: 20.

11. Security MoP: **Annual Report on Drug Control in China, 2009**. In: *Annual Report on Drug Control in China.* Beijing: Ministry of Public Security; 2009: 20.

12. Security MoP: **Annual Report on Drug Control in China, 2010**. In: *Annual Report on Drug Control in China.* Beijing: Ministry of Public Security; 2010: 20.

13. Security MoP: **Annual Report on Drug Control in China, 2011**. In: *Annual Report on Drug Control in China.* Beijing: Ministry of Public Security; 2011: 20.

14. Security MoP: **Annual Report on Drug Control in China, 2012**. In: *Annual Report on Drug Control in China.* Beijing: Ministry of Public Security; 2012: 20.

15. Security MoP: **Annual Report on Drug Control in China, 2013**. In: *Annual Report on Drug Control in China.* Beijing: Ministry of Public Security; 2013: 20.

16. Security MoP: **Annual Report on Drug Control in China, 2014**. In: *Annual Report on Drug Control in China.* Beijing: Ministry of Public Security; 2014: 20.

17. Security MoP: **Annual Report on Drug Control in China, 2015**. In: *Annual Report on Drug Control in China.* Beijing: Ministry of Public Security; 2015: 20.

18. Security MoP: **Annual Report on Drug Control in China, 2016**. In: *Annual Report on Drug Control in China.* Beijing: Ministry of Public Security; 2016: 20.

19. Office CsSCAWC, Group UCW: **2007 Joint Assessment of HIV/AIDS Prevention in China**. In*.*; 2007.

20. Office CsSCAWC, Group UCW: **2004 Joint Assessment of HIV/AIDS Prevention in China**. In*.*; 2004.

21. Ministry of Health PsRoC, UNAIDS, WHO: **2009 Working Report on Estimates of HIV/AIDS Epidemics in China**. In*.* Edited by Ministry of Health PsRoC. Beijing; 2010: 19.

22. Ministry of Health PsRoC, HIV/AIDS JUNPo, Organization WH: **2011 Estimates for the HIV/AIDS Epidemic in China**. In*.* Beijing: Center for Disease Control and Prevention in China; 2011: 21.

23. Zhang F: **Prevalence of HBV and HCV Co-infection and the Impact on the Effectiveness of Antiretroviral Therapy Among HIV/AIDS Patients Receiving Antiretroviral Therapy in China**. In: *the 4th National Chinese Traditional and Western Medicine Conference.* Shanghai; 2012: 44-49.

24. NCAIDS CC: **Update on the AIDS/STD Epidemic in China and Main Response in Control and Prevention in the Second Quarter of 2013**. *Chinese Journal of ADIS and STDs* 2013, **19**(08):545.

25. China MoHotPsRo: **2012 China AIDS Response Progress Report**. In*.*; 2012.

26. National Center for STD/AIDS Prevention and Control CCfDPaC: **Update on the AIDS/STD Epidemics in Chian and Main Responses in Control and Prevention by November 2012**. *Chinese Journal of ADIS and STDs* 2013, **19**(01):1.

27. **HIV/AIDS Prevention Progress in China** [<http://wenku.baidu.com/link?url=X68lIS2PNev3tbHOfPCHQUy2qC37sRN1LZKd9nzrSLbESOuVVkxM92KkCw1VcnN2k9aZ2zrgMuACDJFigEIo6_z03XXNxcn5C8VW6_8Dr6u>]

28. China NHaFPCotPsRo: **2015 China AIDS Response Progress Report**. In*.* Beijing: National Health and Family Planning Commission of the People's Republic of China; 2015: 26.

29. action Et: **HIV and AIDS Data Hub for Asia-Pacific Review in slides: China**. In*.*; 2013.

30. UNAIDS: **HIV Testing in People Who Inject Drugs**. In: *AIDSinfo.* Edited by UNAIDS; 2015.

31. **2011 Prevention Progress on HIV/AIDS and Sexually Transmitted Diseases**. *Chinese Journal of AIDS & STD* 2012(02):64.

32. **2012 Prevention Progress on HIV/AIDS and Sexually Transmitted Diseases**. *Chinese Journal of AIDS & STD* 2013(02):85.

33. **2013 Prevention Progress on HIV/AIDS and Sexually Transmitted Diseases**. *Chinese Journal of AIDS & STD* 2014(02):75.

34. **2014 Prevention Progress on HIV/AIDS and Sexually Transmitted Diseases**. *Chinese Journal of AIDS & STD* 2015(02):87.

35. **2015 Prevention Progress on HIV/AIDS and Sexually Transmitted Diseases**. *Chinese Journal of AIDS & STD* 2016(02):69.

36. **the Ministry of Health Introduce the HIV/AIDS Epidemic Status in China** [<http://news.qq.com/a/20091130/002345.htm>]

37. Cui Y, Guo W, Li D, Wang L, Shi CX, Brookmeyer R, Detels R, Ge L, Ding Z, Wu Z: **Estimating HIV incidence among key affected populations in China from serial cross-sectional surveys in 2010-2014**. *Journal of the International AIDS Society* 2016, **19**(1):20609.

38. **Report on National HIV/AIDS Epidemiology** [<http://news.medlive.cn/all/info-progress/show-71581_171.html>]

39. China CfDCaPi: **Surveillance Data on HIV/AIDS among drug users in China**. In*.* Edited by China CfDCaPi; 2011.

40. Evidencetoaction: **People who inject drugs (2013)**. In*.*; 2013.

41. Wang L, Li D, Ge L, Ding Z, Wang L, Cui Y, Wang N: **HCV Prevalence Among the Populations Under the HIV Sentinel Surveillance Data from 2009-2012 in China**. *Chinese Journal of Epidemiology* 2013, **34**(6):543-547.

42. Qin Q, Smith MK, Wang L, Su Y, Wang L, Guo W, Wang L, Cui Y, Wang N: **Hepatitis C virus infection in China: an emerging public health issue**. *Journal of viral hepatitis* 2015, **22**(3):238-244.

43. Scheinmann R, Hagan H, Lelutiu-Weinberger C, Stern R, Des Jarlais DC, Flom PL, Strauss S: **Non-injection drug use and Hepatitis C Virus: a systematic review**. *Drug and alcohol dependence* 2007, **89**(1):1-12.

44. Xia X, Luo J, Bai J, Yu R: **Epidemiology of hepatitis C virus infection among injection drug users in China: systematic review and meta-analysis**. *Public health* 2008, **122**(10):990-1003.

45. Gao L, Li X, Wang L, Wang C, Luo Y, Zhang X, Qiu G, Wang L: **Characteristics of drug use and sexual behaviors among HIV positive drug users**. *China Preventive Medicine* 2010, **11**(07):702-704.

46. Lin P, Fu X, Li J, Wang Y, Zhong W, Merrill Singer MD, Xu R, Yang F, Liu Y, Zhao Q *et al*: **Characteristics of drug use and sexual behaviors among injecting drug users in Guangdong Province**. *Chinese Journal of ADIS and STDs* 2006, **12**(2):117-122.

47. Yang F, Lin P, Wang Y, He Q, Liu Y, Fu X, Li Y, Long Q: **Logistic regression analysis of factors associated with sharing syringes among injecting drug users in Guangdong**. *Chinese Journal of ADIS and STDs* 2009, **15**(2):143-151.

48. Shen X, Yu A, Bai Y, Yue X, Li J, Yang M, Ren X, Li J: **Factors associated with needle sharing behaviors among intravenous drug users in Gansu Province, China**. *Chinese Journal of Health Statistics* 2012, **29**(4):565-566.

49. Li R, Lan G, Li M, Huang Y, Liu W: **Analysis on impact factors of needle exchange programs among injecting drug users in Guangxi**. *Chinese Journal of ADIS and STDs* 2013, **19**(7):507-513.

50. Wang M, Du J, Huang H, Chen Y, Pan W, Zeng X, Kuang J: **Analysis on Investigation Between Methods of Drug using and Infection of HIV, HCV, HBV and Syphilis of Drug Addicts of Hainan Province**. *China Public Health* 2000, **16**(9):854-855.

51. Qu Z, Wu Z, Shen D, Pu H: **Impact of clean syringe exchange program on prevention of HIV/AIDS among drug users in Hongjiang District, China**. *Practical Preventive Medicine* 2006, **13**(1):85-86.

52. Wang H, Lu C, He Y, Wu J, Hong L, Gao X, Deng X: **Needle Sharing and Its Influential Factors Among Intravenous Drugs users**. *Journal of Tropical Medicine* 2012, **12**(1):22-25.

53. Liang Q, Jiang J, Liu J, Bai Y, Fan Y, Zhang H: **Evaluation of the effect of Australian agency for international development funded project among injection drug users in Liuzhou City**. *Chinese Journal of Disease Control and Prevention* 2011, **15**(11):956-958.

54. Huang L, Feng T, Liu D, Chen L, Wang Q, Wang X: **Analysis on needle-sharing and sex-related risk behaviors among drug users in Shenzhen city**. *Chinese Journal of Public Health* 2005, **21**(7):859-860.

55. He Y, Wang Y, Ma Z, Ruan Y, Liu S, Zhou F, Zhang L, Chen K, Yin L, Qin G *et al*: **Survey on sharing injection equiment incidence among drug users in Liangshan of Sichuan Province**. *Chinese Journal of Public Health* 2005, **21**(11):1283-1285.

56. Lai W, Qiao L, Deng B, Wen J, Zhang X, Zhang L: **High Risk Behaviors of AIDS in Needle Using Among Injection Drug Users in 10 Counties of Sichuan**. *Journal of Preventive Medicine Information* 2009, **25**(6):392-394.

57. Zhou Z, Tan A, Huang L, Chen W: **Analysis on high risk behavior of needle sharing and its influence factors among drug abusers**. *Chinese Journal of Public Health* 2007, **23**(9):1043-1044.

58. Li R, Lan G, Li M, Huang Y, Li W, Yang J: **Influence of Needle Exchange Programs on Injection Risk Behaviors and HIV Infection Among Injection Drug User**. *Practical Preventive Medicine* 2013, **20**(8):925-927.

59. Ming Z, Wu Z, Liu W, Liang S, Lu W, Zhou Y: **Effectiveness of Needle Exchange Combined with Peer Education Among IDUs in Guangxi**. *Chinese Journal of ADIS and STDs* 2005, **11**(3):188-190.

60. Cheng F, Chen H, Li J, Zhang L, Hu H, Zhang J, Cheng H, Stewart W, Shen J: **SASH survey on high risk behaviors of IDUs in four cities of Yunnan and Sichuan**. *Chinese Journal of Drug Dependence* 2003, **12**(4):294-298.

61. Abdala N, Gleghorn AA, Carney JM, Heimer R: **Can HIV-1-contaminated syringes be disinfected? Implications for transmission among injection drug users**. *Journal of acquired immune deficiency syndromes (1999)* 2001, **28**(5):487-494.

62. Newmeyer JA: **Why bleach? Fighting AIDS contagion among intravenous drug users: the San Francisco experience**. *Journal of psychoactive drugs* 1988, **20**(2):159-163.

63. Froner GA, Rutherford GW, Rokeach M: **Injection of sodium hypochlorite by intravenous drug users**. *Jama* 1987, **258**(3):325.

64. Dou Y, Tong G: **Why the proportion of detoxification is as low as 2%?** In*.*; 2005.

65. Fan J, Zhan L: **Effectiveness and Progress of Methadone Maintenance Treatment in China**. *Guizhou Medicine and Pharmacy* 2013, **37**(12):1122-1126.

66. Zhang L, Chow EP, Zhuang X, Liang Y, Wang Y, Tang C, Ling L, Tucker JD, Wilson DP: **Methadone maintenance treatment participant retention and behavioural effectiveness in China: a systematic review and meta-analysis**. *PloS one* 2013, **8**(7):e68906.

67. Cao X, Pang L, Rou K, Yin W, Wang C, Luo W, Mi G, Li J, Wu Z, Group MMTNW: **Five-year cohort study of the retention rate and its impact factors for 1057 patients receiving methadone maintenance treatment at the first 8 community-based clinics in China**. *Chinese Journal of ADIS and STDs* 2010, **16**(3):211-225.

68. Zeng T, Ma L, Wang W, Zhang Z, Cao W: **Comparison and Analysis on Performance of Methadone Maintenance Treatment Among Seven Provinces in China From 2007-2008**. *Modern Preventive Medicine* 2010, **37**(13):2521-2525+2530.

69. Liu E, Liang T, Shen L, Zhong H, Wang B, Wu Z, Detels R: **Correlates of methadone client retention: a prospective cohort study in Guizhou province, China**. *The International journal on drug policy* 2009, **20**(4):304-308.

70. Tao L, Liang W, Liu H: **Study on the transition progress from snorting to injection**. *Chinese Journal of Drug Dependence* 2000, **9**(4):300-302.

71. Xiao Y: **The investigation of influence of the manner change factors in drug addicts analysis**. Kunming: Kunming Medical College; 2010.

72. Li Y, Xiao L, Xie K, Li Y, Chen Q, Yan K, Zhang H, Zhang G, Luo X: **A Retrospective Study on Risk Factors of Ways of Drug Use and its Transition**. In: *The Teenth China Medical Board Academic Conference on Psychiatry Medical: 2012; Nanjing, Jiangsu Province*; 2012: 1.

73. Liao Y, Jiang J, Wei S, Li M, Shi S, He B, Xiang S, Deng W, Ning C, Xu N *et al*: **Changes in Sexual Behaviors After Drug Use and Their Influencing Factors Among Male Drug Users in Guangxi, China**. *Chinese Journal of Disease Control and Prevention* 2012, **16**(12):1032-1035.

74. Zhuang X, Wang Y, Chow EP, Liang Y, Wilson DP, Zhang L: **Risk factors associated with HIV/HCV infection among entrants in methadone maintenance treatment clinics in China: a systematic review and meta-analysis**. *Drug and alcohol dependence* 2012, **126**(3):286-295.

75. Baggaley Rf Fau - Boily M-C, Boily Mc Fau - White RG, White Rg Fau - Alary M, Alary M: **Risk of HIV-1 transmission for parenteral exposure and blood transfusion: a systematic review and meta-analysis**. (0269-9370 (Print)).

76. Boily MC, Baggaley RF, Wang L, Masse B, White RG, Hayes RJ, Alary M: **Heterosexual risk of HIV-1 infection per sexual act: systematic review and meta-analysis of observational studies**. *The Lancet Infectious diseases* 2009, **9**(2):118-129.

77. Xia YH, Chen W, Tucker JD, Wang C, Ling L: **HIV and hepatitis C virus test uptake at methadone clinics in Southern China: opportunities for expanding detection of bloodborne infections**. *BMC public health* 2013, **13**:899.

78. Zheng Y, Qi A: **Estimation of HIV diagnosis among HIV infected patients**. *Chinese Journal of Hospital Statistics* 1997, **4**(3):190-192.

79. Vlahov D, Celentano DD: **Access to highly active antiretroviral therapy for injection drug users: adherence, resistance, and death**. *Cadernos de saude publica* 2006, **22**(4):705-718.

80. Huan Z, Fuzhi W, Lu L, Min Z, Xingzhi C, Shiyang J: **Comparisons of Adherence to Antiretroviral Therapy in a High-Risk Population in China: A Systematic Review and Meta-Analysis**. *PloS one* 2016, **11**(1):e0146659.

81. Zhao Y, Shi CX, McGoogan JM, Rou K, Zhang F, Wu Z: **Methadone maintenance treatment and mortality in HIV-positive people who inject opioids in China**. *Bulletin of the World Health Organization* 2013, **91**(2):93-101.

82. Ma Y: **Virologic outcome and their predictors in HIV infected adults receiving long term first-line antiretroviral treatment in 8 provinces in China**. Beijing: National Center for Disease Control and Prevention; 2012.

83. Dou Z, Zhang F, Zhao Y, Jin C, Zhao D, Gan X, Ma Y: **[Progress on China' s national free antiretroviral therapy strategy in 2002-2014]**. *Zhonghua liu xing bing xue za zhi = Zhonghua liuxingbingxue zazhi* 2015, **36**(12):1345-1350.

84. Zhang F, Dou Z, Ma Y, Zhao Y, Liu Z, Bulterys M, Y.Chen R: **Five-year Outcomes of the China National Free Antiretroviral Treatment Program**. *Annual Internal Medicine* 2009, **151**(4):1-42.

85. Reddon H, Milloy MJ, Simo A, Montaner J, Wood E, Kerr T: **Methadone maintenance therapy decreases the rate of antiretroviral therapy discontinuation among HIV-positive illicit drug users**. *AIDS and behavior* 2014, **18**(4):740-746.

86. Kwon JA, Anderson J, Kerr CC, Thein HH, Zhang L, Iversen J, Dore GJ, Kaldor JM, Law MG, Maher L *et al*: **Estimating the cost-effectiveness of needle-syringe programs in Australia**. *AIDS (London, England)* 2012, **26**(17):2201-2210.

87. Patz JA, Jodrey D: **Occupational health in surgery: risks extend beyond the operating room**. *The Australian and New Zealand journal of surgery* 1995, **65**(9):627-629.

88. Short LJ, Bell DM: **Risk of occupational infection with blood-borne pathogens in operating and delivery room settings**. *American journal of infection control* 1993, **21**(6):343-350.

89. Gerberding JL: **Management of occupational exposures to blood-borne viruses**. *The New England journal of medicine* 1995, **332**(7):444-451.

90. MacDonald M, Crofts N, Kaldor J: **Transmission of hepatitis C virus: rates, routes, and cofactors**. *Epidemiologic reviews* 1996, **18**(2):137-148.

91. Kiyosawa K, Sodeyama T, Tanaka E, Nakano Y, Furuta S, Nishioka K, Purcell RH, Alter HJ: **Hepatitis C in hospital employees with needlestick injuries**. *Annals of internal medicine* 1991, **115**(5):367-369.

92. Sodeyama T, Kiyosawa K, Urushihara A, Matsumoto A, Tanaka E, Furuta S, Akahane Y: **Detection of hepatitis C virus markers and hepatitis C virus genomic-RNA after needlestick accidents**. *Archives of internal medicine* 1993, **153**(13):1565-1572.

93. Hamid SS, Farooqui B, Rizvi Q, Sultana T, Siddiqui AA: **Risk of transmission and features of hepatitis C after needlestick injuries**. *Infection control and hospital epidemiology* 1999, **20**(1):63-64.

94. De Carli G, Puro V, Ippolito G: **Risk of hepatitis C virus transmission following percutaneous exposure in healthcare workers**. *Infection* 2003, **31 Suppl 2**:22-27.

95. Kwon JA, Iversen J, Maher L, Law MG, Wilson DP: **The impact of needle and syringe programs on HIV and HCV transmissions in injecting drug users in Australia: a model-based analysis**. *Journal of acquired immune deficiency syndromes (1999)* 2009, **51**(4):462-469.

96. Vickerman P, Hickman M, Judd A: **Modelling the impact on Hepatitis C transmission of reducing syringe sharing: London case study**. *International journal of epidemiology* 2007, **36**(2):396-405.

97. Cook PA, McVeigh J, Syed Q, Mutton K, Bellis MA: **Predictors of hepatitis B and C infection in injecting drug users both in and out of drug treatment**. *Addiction (Abingdon, England)* 2001, **96**(12):1787-1797.

98. Hayashi K, Montaner J, Kaplan K, Suwannawong P, Wood E, Qi J, Kerr T: **Low uptake of hepatitis C testing and high prevalence of risk behavior among HIV-positive injection drug users in Bangkok, Thailand**. *Journal of acquired immune deficiency syndromes (1999)* 2011, **56**(5):e133-135.

99. Lazarus JV, Sperle I, Maticic M, Wiessing L: **A systematic review of Hepatitis C virus treatment uptake among people who inject drugs in the European Region**. *BMC Infect Dis* 2014, **14 Suppl 6**:S16.

100. Kamal SM, Moustafa KN, Chen J, Fehr J, Abdel Moneim A, Khalifa KE, El Gohary LA, Ramy AH, Madwar MA, Rasenack J *et al*: **Duration of peginterferon therapy in acute hepatitis C: a randomized trial**. *Hepatology (Baltimore, Md)* 2006, **43**(5):923-931.

101. Malnick SD, Basevitch A: **Peginterferon alfa-2b therapy in acute hepatitis C: impact of onset of therapy on sustained virologic response**. *Gastroenterology* 2006, **131**(2):683; author reply 683-684.

102. Villano SA, Nelson KE, Vlahov D, Purcell RH, Saah AJ, Thomas DL: **Hepatitis A among homosexual men and injection drug users: more evidence for vaccination**. *Clinical infectious diseases : an official publication of the Infectious Diseases Society of America* 1997, **25**(3):726-728.

103. **Recommendations for follow-up of health-care workers after occupational exposure to hepatitis C virus**. *MMWR Morbidity and mortality weekly report* 1997, **46**(26):603-606.

104. Hadziyannis SJ, Sette H, Jr., Morgan TR, Balan V, Diago M, Marcellin P, Ramadori G, Bodenheimer H, Jr., Bernstein D, Rizzetto M *et al*: **Peginterferon-alpha2a and ribavirin combination therapy in chronic hepatitis C: a randomized study of treatment duration and ribavirin dose**. *Annals of internal medicine* 2004, **140**(5):346-355.

105. Alfaleh FZ, Hadad Q, Khuroo MS, Aljumah A, Algamedi A, Alashgar H, Al-Ahdal MN, Mayet I, Khan MQ, Kessie G: **Peginterferon alpha-2b plus ribavirin compared with interferon alpha-2b plus ribavirin for initial treatment of chronic hepatitis C in Saudi patients commonly infected with genotype 4**. *Liver international : official journal of the International Association for the Study of the Liver* 2004, **24**(6):568-574.

106. Bosques-Padilla F, Trejo-Estrada R, Campollo-Rivas O, Cortez-Hernandez C, Dehesa-Violante M, Maldonado-Garza H, Perez-Gomez R, Cabrera-Valdespino A: **Peginterferon alfa-2a plus ribavirin for treating chronic hepatitis C virus infection: analysis of Mexican patients included in a multicenter international clinical trial**. *Annals of hepatology* 2003, **2**(3):135-139.

107. **Death rate** [<http://data.worldbank.org/indicator/SP.DYN.CDRT.IN>]

108. Cao X, Wu Z, Li L, Pang L, Rou K, Wang C, Luo W, Yin W, Li J, McGoogan JM: **Mortality among methadone maintenance clients in China: a six-year cohort study**. *PloS one* 2013, **8**(12):e82476.

109. Zheng X, Zhang J, Wang X, Duan S, Qu S, Duan Y, Zhang G: **[The natural history of HIV infection among IDUs in Ruili, Yunnan province, China]**. *Zhonghua liu xing bing xue za zhi = Zhonghua liuxingbingxue zazhi* 2000, **21**(1):17-18.

110. Liu E, Rou K, McGoogan JM, Pang L, Cao X, Wang C, Luo W, Sullivan SG, Montaner JS, Bulterys M *et al*: **Factors associated with mortality of HIV-positive clients receiving methadone maintenance treatment in China**. *The Journal of infectious diseases* 2013, **208**(3):442-453.

111. Jiang H, Xie N, Cao B, Tan L, Fan Y, Zhang F, Yao Z, Liu L, Nie S: **Determinants of progression to AIDS and death following HIV diagnosis: a retrospective cohort study in Wuhan, China**. *PloS one* 2013, **8**(12):e83078.

112. Masiira B, Baisley K, Mayanja BN, Kazooba P, Maher D, Kaleebu P: **Mortality and its predictors among antiretroviral therapy naive HIV-infected individuals with CD4 cell count >/=350 cells/mm(3) compared to the general population: data from a population-based prospective HIV cohort in Uganda**. *Global health action* 2014, **7**:21843.

113. Ledergerber B, Lundgren JD, Walker AS, Sabin C, Justice A, Reiss P, Mussini C, Wit F, d'Arminio Monforte A, Weber R *et al*: **Predictors of trend in CD4-positive T-cell count and mortality among HIV-1-infected individuals with virological failure to all three antiretroviral-drug classes**. *Lancet (London, England)* 2004, **364**(9428):51-62.

114. Mathers BM, Degenhardt L, Bucello C, Lemon J, Wiessing L, Hickman M: **Mortality among people who inject drugs: a systematic review and meta-analysis**. *Bulletin of the World Health Organization* 2013, **91**(2):102-123.

115. Kielland KB, Skaug K, Amundsen EJ, Dalgard O: **All-cause and liver-related mortality in hepatitis C infected drug users followed for 33 years: a controlled study**. *J Hepatol* 2013, **58**(1):31-37.

116. Smit C, van den Berg C, Geskus R, Berkhout B, Coutinho R, Prins M: **Risk of hepatitis-related mortality increased among hepatitis C virus/HIV-coinfected drug users compared with drug users infected only with hepatitis C virus: a 20-year prospective study**. *Journal of acquired immune deficiency syndromes (1999)* 2008, **47**(2):221-225.

117. Pearlman BL, Traub N: **Sustained virologic response to antiviral therapy for chronic hepatitis C virus infection: a cure and so much more**. *Clinical infectious diseases : an official publication of the Infectious Diseases Society of America* 2011, **52**(7):889-900.

118. **Global Burden of Disease Study 2016** [<http://ghdx.healthdata.org/gbd-2016>]

119. Salomon JA, Haagsma JA, Davis A, de Noordhout CM, Polinder S, Havelaar AH, Cassini A, Devleesschauwer B, Kretzschmar M, Speybroeck N *et al*: **Disability weights for the Global Burden of Disease 2013 study**. *Lancet Glob Health* 2015, **3**(11):e712-723.

120. Stanaway JD, Flaxman AD, Naghavi M, Fitzmaurice C, Vos T, Abubakar I, Abu-Raddad LJ, Assadi R, Bhala N, Cowie B *et al*: **The global burden of viral hepatitis from 1990 to 2013: findings from the Global Burden of Disease Study 2013**. *Lancet* 2016, **388**(10049):1081-1088.

121. Shi J, Zhao LY, Epstein DH, Zhao C, Shuai Y, Yan B, Jin J, Lu L: **The effect of methadone maintenance on illicit opioid use, human immunodeficiency virus and hepatitis C virus infection, health status, employment, and criminal activity among heroin abusers during 6 months of treatment in china**. *Journal of addiction medicine* 2007, **1**(4):186-190.

122. Barrio G, De La Fuente L, Lew C, Royuela L, Bravo MJ, Torrens M: **Differences in severity of heroin dependence by route of administration: the importance of length of heroin use**. *Drug and alcohol dependence* 2001, **63**(2):169-177.

123. Liu J, Ling L, Zhao L, Lin P, He Q: **Evaluation and Decision Analysis on Community-based Methadone Maintenance Treatment in Guangdong**. *Chinese Journal of Health Statistics* 2007, **24**(5):501-504.

124. Chen W, Ling L, He Q, Chen A, Chen J, Xia Y: **Performance Evaluation and Policy Recommendations on Community Methadone Maintenance Treatment in Guangdong Province**. *Chinese Journal of Health Policy* 2010, **3**(3):39-44.

125. Liu K, Wang M: **The Cost Benefit Analysis Between Methadone Maintenance Treatment and Compulsory Detoxification**. *Chinese Journal of Health Policy* 2010, **3**(2):30-33.

126. Lin SH, Chen KC, Lee SY, Hsiao CY, Lee IH, Yeh TL, Chen PS, Lu RB, Yang YK: **The economic cost of heroin dependency and quality of life among heroin users in Taiwan**. *Psychiatry research* 2013, **209**(3):512-517.

127. Li X: **Study on the Effect and Economic Evaluation of Community Methadone Maintenance Treatment for Patients with Heroin Dependence**. *Doctor.* 中南大学; 2007.

128. Zhang Y, Cui Y, Gao H, Liu J: **Analysis on Fund Demand in Poor Areas with Low HIV/AIDS Prevalence**. *Chinese Journal of Disease Prevention and Control* 2009, **13**(04):499-501.

129. Duan J, Qi T, Li S: **Cost-effectiveness of Screening Test among Blood Donors**. *Chinese Journal of Blood Transfusion* 2011, **24**(01):65-66.

130. Yue Y, Liu J, Zheng Y, Hu X, Xie H, Bai S: **Cost-effectiveness Analysis on the HIV Voluntary Counseling and Testing in Xinjiang**. *Journal of Xinjiang Medical University* 2013, **36**(02):241-244.

131. Guo Z: **The direct medical costs analysis of national free antiretroviral treatment for HIV/AIDS patients**. *Master.* China Xiehe Medical College; 2008.

132. Qiu X, Ming D: **Cost-effectiveness Analysis on Three Testing Methods of Hepatitis C**. *International Journal Laboratory Medicine* 2008, **29**(11):1048.

133. Ma Y, Ha N, Yang X: **Pharmacoeconomic Evaluation of 2 Therapeutic Schemes for Chronic Hepatitis C**. *China Pharmacy* 2010, **21**(18):1642-1645.

134. Xing Y, Sun J, Cao W, Lee L, Guo H, Li H, Duan S: **Economic evaluation of methadone maintenance treatment in HIV/AIDS control among injecting drug users in Dehong, China**. *AIDS care* 2012, **24**(6):756-762.
